# Development and External Validation of the Cantonese Dietary Index: A Population-Based Approach to Assess Diet Quality and Metabolic Risk

**DOI:** 10.3390/nu18111678

**Published:** 2026-05-24

**Authors:** Yue Xi, Shunming Zhang, Xinyue Wang, Rong Luo, Bin Deng, Wei Hu, Wenhua Ling, Kaijun Niu, Huilian Zhu, Yuming Chen

**Affiliations:** 1 Department of Epidemiology, School of Public Health, Sun Yat-sen University, Guangzhou 510080, China; xiyue5@mail2.sysu.edu.cn (Y.X.); wangxy729@mail2.sysu.edu.cn (X.W.); huwei59@mail2.sysu.edu.cn (W.H.); 2 Guangdong Nutrition Society, Guangzhou 510080, China; lingwh@mail.sysu.edu.cn; 3 School of Public Health, Xi’an Jiaotong University Health Science Center, Xi’an 710061, China; zhangshunming@xjtu.edu.cn; 4 Amway (China) Co., Ltd., Guangzhou 510700, China; rose_luo@amway.com (R.L.); ben.deng@amway.com (B.D.); 5 Department of Nutrition, School of Public Health, Sun Yat-sen University, Guangzhou 510080, China; 6 School of Public Health, Tianjin University of Traditional Chinese Medicine, Tianjin 301617, China

**Keywords:** dietary index, Cantonese diet, Eastern dietary patterns, dietary quality, metabolic syndrome, nutritional epidemiology

## Abstract

**Objectives**: We aimed to develop a practical dietary quality score reflecting the Cantonese dietary pattern and evaluate its validity against established indices. **Methods**: The Cantonese Dietary Index (CDI, 岭南膳食指数) was constructed based on Cantonese dietary principles. Reliability was assessed using intraclass correlation coefficients (ICC) over 5–6 years in the GNHS. Validity was evaluated using Spearman correlations with dietary indices (aMed, DASH, and DBI) and by comparing associations with metabolic syndrome (MetS) across dietary indices using regression models. The CDI was developed from the Guangzhou Nutrition and Health Study cohort (GNHS) and validated in the Tianjin Chronic Low-grade Systemic Inflammation and Health (TCLSIH) cohort and the National Health and Nutrition Examination Survey (NHANES). **Results**: A total of 4025 (GNHS), 29,165 (TCLSIH), and 28,890 (NHANES) participants were included. Median CDI scores were 58.5, 51.0, and 49.0, respectively. The 5–6-year ICC was 0.33 (*p* < 0.001). The CDI was moderately correlated with dietary indices across the three studies (GNHS: from −0.55 [DBI-LBS] to 0.61 [DASH], TCLSIH: from −0.61 [DBI-DQD] to 0.71 [DASH], NHANES: from −0.33 [DBI-DQD] to 0.68 [DASH]). The odds ratios (95% CIs) of MetS for CDI, aMed, and DASH scores were 0.80 (0.74, 0.86), 0.91 (0.84,0.99), and 0.83 (0.77, 0.90) in GNHS, 0.95 (0.92, 0.98), 0.99 (0.96, 1.02), and 0.92 (0.89, 0.95) in TCLSIH, and 0.80 (0.77, 0.84), 0.80 (0.76, 0.84), and 0.72 (0.69, 0.76) in NHANES. **Conclusions**: The CDI demonstrated moderate validity and reliability in Chinese populations and was inversely associated with MetS.

## 1. Introduction

The Cantonese dietary pattern is considered a healthy Eastern dietary pattern [1] characterized by a wide variety of ingredients, abundant intake of fruits and vegetables, sufficient consumption of aquatic products, a light flavor, high tea consumption, lower alcohol intake, and an emphasis on nutritional balance. Previous studies have suggested that morbidity and mortality rates for chronic diseases, such as cardiovascular disease (CVD) and cancer, are lower in Guangdong than in other regions of China and Western countries [2,3,4,5,6]. As of 2024, the population in the Lingnan region, which generally includes Guangdong, Guangxi, and Hainan Provinces, has reached approximately 188 million [7], and the number of individuals adhering to this dietary pattern may be even larger [6,8]. Given the critical role of diet in human health [9], promoting this regional dietary pattern may contribute to disease prevention and lifelong health. However, a standardized index for assessing adherence to this dietary pattern has not yet been developed.

Diet quality indices, consisting of a combination of foods and nutrient components that reflect dietary guidelines or recommendations [10,11], are widely used [12,13]. This is because a single food cannot capture overall dietary intake, and dietary patterns are difficult to assess. A dietary index can assess the extent to which eating patterns align with dietary guidelines, evaluate individual adherence to a specific diet [14], monitor temporal trends in population-based studies and national surveys [14,15,16,17], and assess the effectiveness of dietary patterns in preventing chronic diseases [13,15,18,19,20]. Among them, the Mediterranean diet score (MED), Dietary Approaches to Stop Hypertension (DASH) score, and Healthy Eating Index (HEI) are the most widely used indices and have been suggested to be associated with lower risks of type 2 diabetes mellitus (T2DM), hypertension, CVD, and cancer [21,22,23,24]. However, most dietary indices are derived from Western dietary patterns, and their applicability to Chinese or other non-Western diets is limited due to cultural and regional differences [20,25]. Existing indices based on Chinese diets, like the Diet Balance Index (DBI) [26], the China Dietary Guidelines Index (CDGI) [27], and the Chinese Food Pagoda Score (CFPS) [28], are relatively complex to calculate in large-scale epidemiological research and may not be suitable since Chinese residents are presently undergoing unprecedented changes in dietary structures, with these becoming increasingly complex and diverse. The dietary indices focused on Eastern dietary patterns remain scarce, especially in recent decades. There is an urgent need to develop a novel dietary index based on the Cantonese dietary pattern.

Therefore, this study aims to: (1) develop a simple and practical dietary scoring system, the Cantonese Dietary Index (CDI, 岭南膳食指数), based on the Guangdong dietary pattern, (2) examine the associations of the CDI with metabolic syndrome and its components, and (3) validate the CDI in three large-scale cohort studies for generalizability.

## 2. Materials and Methods

### 2.1. Study Design and Sample

This cross-sectional study for CDI development was based on the Guangzhou Nutrition and Health Study (GNHS) cohort in southern China, with external validation conducted using the Tianjin Chronic Low-grade Systemic Inflammation and Health (TCLSIH) cohort in northern China, and the National Health and Nutrition Examination Survey (NHANES).

The GNHS is a population-based prospective study that enrolled 4048 healthy adults aged 40–75 years who had lived in Guangzhou for more than 5 years between 2008 and 2013. Participants were followed up about every 3 years, with a median duration of 9.48 years [29]. The TCLSIH cohort is an ongoing prospective study initiated in May 2013, including general adults aged ≥18 years who had lived in Tianjin for at least 5 years, with follow-up every 1~2 years [30]. NHANES is a national survey led by the Centers for Disease Control and Prevention (CDC) [31] since 1999. All NHANES data and documentation related to the study are publicly accessible. The application of the CDI in NHANES was intended to assess its generalizability beyond the original population, as a robust dietary index should not only reflect local dietary characteristics but also align with general dietary principles and show consistent associations with health outcomes across different populations.

Participants in all studies underwent health examinations and completed questionnaires on diet, lifestyle factors, and disease history. All procedures were conducted in accordance with the principles of the Declaration of Helsinki. The GNHS (SYSU-GWYL2018048, 17 July 2018) and TCLSIH (TMUhMEC 201430, 28 October 2014) were approved by the Institutional Review Boards of Sun Yat-sen University and Tianjin Medical University, respectively. NHANES was approved by the National Center for Health Statistics Research Ethics Review Board for each survey cycle (2005–2016). All participants had written informed consent [31].

Participants were excluded if they: (1) had incomplete dietary data, (2) had mental disorders, cognitive impairment, disability, or malignant diseases, (3) had implausible energy intake (male: <800 kcal/d or >4200 kcal/d, female: <500 kcal/d or >3500 kcal/d). The final sample included 4025 participants in GNHS, 29,165 in TCLSIH, and 28,890 in NHANES (2005–2016). Supplementary Figure S1 shows the study participant selection process.

### 2.2. Dietary Assessment

In the two Chinese cohorts, usual food intake over the past year was assessed using validated semi-quantitative food frequency questionnaires (FFQs) [30,32]. Total energy and nutrient intake were calculated according to the Chinese Food Composition Tables [33]. In NHANES, dietary intake was assessed using two non-consecutive 24-h dietary recalls, and nutrient and energy intake were estimated using the USDA Food and Nutrient Database [31]. For GNHS, the average consumption from baseline and the 1st follow-up was calculated for analysis. For TCLSIH, the baseline intake was used. For NHANES, the average intake during the 2-day survey was calculated. For the reliability analyses, dietary data from the third GNHS follow-up were used.

### 2.3. Summary of Cantonese Dietary Patterns and Assessment of Diet Quality

#### 2.3.1. Calculation of Cantonese Dietary Index

The CDI was developed based on eight core components of the Cantonese dietary pattern [34], the Chinese Dietary Guidelines (2022) [35], and existing indices (DASH [36], and aMed [37]). The CDI conceptualizes diet as a multidimensional construct, including dietary intake and habitual eating behaviors. Accordingly, the CDI comprises two domains: dietary consumption and eating behaviors.

Components were selected based on the eight core principles, the Chinese Dietary Guidelines (2022), and expert consensus. The Chinese Dietary Guidelines (2022) were used to define recommended intake levels and determine scoring direction (i.e., adequacy or moderation), while expert input ensured the cultural relevance and contextual appropriateness. The final CDI comprises 18 components (detailed definitions are provided in the Supplementary Methods: Definitions on Components of CDI).

Domain 1 (dietary consumption): fresh vegetables, dark-colored vegetables, fruits, total animal foods, the ratio of aquatic and poultry to total animal foods, dairy products, whole grains and miscellaneous beans, soybeans and nuts, saturated fat, salt, and added sugars.

Domain 2 (eating behaviors): dietary diversity, breakfast frequency, tea consumption, alcohol consumption, frequency of fried and preserved food intake, food–medicine homologous substances consumption, and dietary supplement use.

We further classified CDI components as general diet-quality components, Cantonese culture-specific markers, or components with both nutritional and cultural relevance. This classification is now presented in the Supplementary Methods (Domain, main role, and rationale of eight core components of the Cantonese dietary pattern) to make the theoretical framework more transparent.

Regarding DASH [36] and aMED [37], the dietary scores most strongly associated with disease [20], the CDI was designed according to populations’ quintile ranking for dietary consumption items and median values or two categorical choices for most eating behavior items. The scoring of CDI was based on the energy-density-adjusted consumptions of food or nutrients (amounts of foods or nutrients per 1000 kcal). In addition, to improve applicability, we further refined the scoring criteria based on the quantitative recommendations of the Chinese Dietary Guidelines (2022) and added an alternative “absolute intake” scoring approach based on specific intake levels in GNHS and NHANES. As for NHANES, dietary intakes were quantified by converting FPED components into gram equivalents based on predefined conversion factors (Supplementary Methods: Conversion of FPED food pattern components to gram equivalents), which were subsequently used for dietary scoring. Considering the multidimensional nature of the dietary pattern and the varying contributions of its components to health, unequal weights were assigned based on the core principles of the Cantonese dietary pattern, the Chinese Dietary Guidelines (2022), and expert opinion. The total CDI score was calculated by summing all items’ scores, with a maximum total score of 100 points. A higher score indicates better adherence to Cantonese dietary patterns and higher diet quality. Table 1 shows the detailed criteria for calculating the CDI using both the quintile-based approach and the absolute intake method.

#### 2.3.2. Calculation of Other Diet-Quality Scores

DASH [36], aMed [20,37,38,39], and one of the Chinese official indices, DBI [26], were selected as reference indices for comparison. Scoring details are provided in Supplementary Methods (Scoring details of aMed, DASH, and DBI).

### 2.4. Outcome Assessment

Metabolic syndrome and its components were the main outcomes. According to the International Diabetes Federation (IDF) global consensus on metabolic syndrome, metabolic syndrome was diagnosed when three or more of the following criteria were met [40]:

(1) Abdominal obesity: Waist circumference ≥ 90 cm in men or ≥80 cm in women.
(2) Hyperglycemia: Fasting blood glucose ≥ 5.6 mmol/L or a diagnosis of type 2 diabetes.
(3) Hypertension: SBP ≥ 130 mmHg or DBP ≥ 85 mmHg, or a diagnosis of hypertension.
(4) High TG: TG ≥ 1.70 mmol/L or specific treatment for elevated TG.
(5) Low HDL-C: HDL-C < 1.03 mmol/L in men or <1.29 mmol/L in women.

In GNHS and TCLSIH, the first follow-up and baseline data were used, respectively. In NHANES, analyses were limited to participants with fasting blood samples.

### 2.5. Assessment of Covariates

Data on age, sex, household income per month, educational attainments, marital status, smoking status, physical activity, dietary supplement use, personal history of disease and medication use, and total energy intake were collected using standardized questionnaires. Detailed information is described in Supplementary Methods (Covariables collection).

### 2.6. Statistical Analysis

The Kolmogorov–Smirnov test and Q-Q plots assessed the normality of the continuous variables. Characteristics were summarized as medians (IQR) for continuous variables and percentages for categorical variables.

The final CDI scoring and weighting scheme was specified before examining associations with metabolic syndrome. Sensitivity analyses in GNHS tested alternative weighting schemes by increasing or decreasing selected component weights to examine the robustness of the predefined weighting scheme and were not used to select the final CDI version. The results of the sensitivity analyses are presented in Supplementary Table S1.

#### 2.6.1. Reliability Analyses

Reliability of the CDI was assessed in GNHS using Cronbach’s alpha coefficients, Spearman partial correlations, and intra-class correlation coefficients (ICC). Cronbach’s alpha coefficients were calculated as a descriptive measure of internal consistency. Given that the CDI is a multidimensional index designed to capture diverse dietary components and behaviors, high internal consistency was not expected and is not a primary criterion for evaluating the index’s reliability. Partial correlations adjusted for age and sex, along with intra-class correlation coefficients, which represent an important dimension of reliability in nutritional epidemiology, were used to assess the long-term stability of CDI total scores over a 5–6-year period.

#### 2.6.2. Validity Analyses

The validity of the CDI was evaluated using multiple approaches. Content validity was assessed by correlations between the CDI and nutrient intake. Convergent validity was evaluated by comparing the CDI with established dietary indices (aMed, DASH, and DBI). Construct validity was assessed by examining the associations between the CDI and metabolic syndrome and its components. Binary logistic models were used to estimate odds ratios (ORs) and 95% confidence intervals (CIs) for CDI and other diet-quality scores (both continuous and tertile variables) in relation to MetS and its components across three studies. All indices were standardized (per standard deviation increase). The adjusted covariates were sequentially included: age, sex (Model 1), educational attainment, marital status, smoking status, physical activity, antihypertensive medications, lipid-lowering medications, antidiabetic medications, and total energy intake (Model 2). Dietary supplements were adjusted as a covariate in models for other diet-quality scores. Linear trend tests were conducted using median tertile values as continuous exposures. Multicollinearity was assessed using variance inflation factors, and the results indicated acceptable collinearity (all variance inflation factors were ≤2.5). The restricted cubic spline with 3 knots at the 25th, 50th, and 75th percentiles of the CDI was used to examine dose–response relationships.

All statistical analyses were conducted using R version 4.5.1. A two-sided *p* < 0.05 was considered statistically significant.

## 3. Results

### 3.1. Characteristics of Participants

The characteristics of participants across the three cohorts are presented in Table 2. The median age was 58.0 (54.0, 62.0) years in the GNHS cohort, 42.6 (33.6, 52.6) years in the TCLSIH cohort, and 49.0 (35.0, 64.0) years in the NHANES study. Females accounted for 68.2% of participants in GNHS and 51.3% in NHANES, whereas males accounted for 55.4% in TCLSIH. Compared with participants in the other two cohorts, those in the GNHS cohort were more likely to be married, had lower educational attainment, and had lower proportions of smoking and drinking. The GNHS cohort also had a higher prevalence of hypertension and elevated triglyceride levels. Detailed characteristics stratified by CDI tertiles are presented in Supplementary Table S2.

### 3.2. The Dietary Quality Scores of the Participants

The median total CDI scores were 58.5 (52.0, 64.5) in the GNHS cohort, 51.0 (44.5, 57.5) in the TCLSIH cohort, and 49.0 (41.0, 57.5) in the NHANES study (Supplementary Table S3). The corresponding ranges of CDI scores were 27.0~84.5 in GNHS, 15.0~85.0 in TCLSIH, and 14.0~89.0 in NHANES. Across CDI facets, participants in the GNHS cohort had higher scores in facets 2, 6, and 8 and lower scores in facet 1 than participants in the other cohorts. Detailed component scores stratified by CDI tertiles and sex are presented in Supplementary Table S4.

### 3.3. Reliability of the CDI in the GNHS Cohort

**Figure 1:**
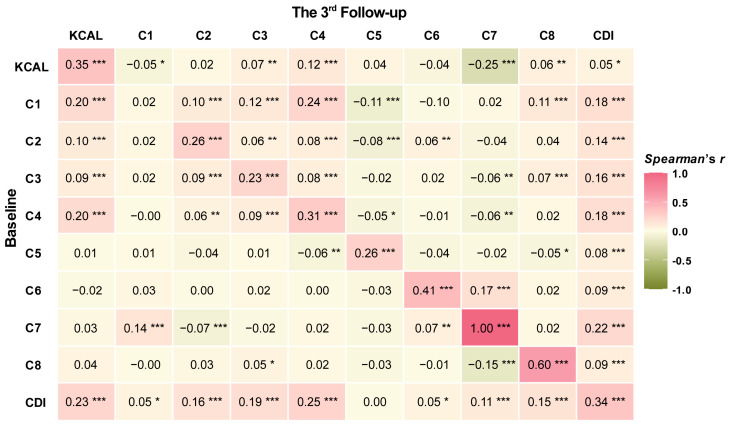
Heatmap of partial Spearman’s correlations of CDI scores between the baseline and the 3rd follow-up in the GNHS. Partial Spearman’s correlations were adjusted for sex and age. Colors in the heatmap represent the direction and magnitude of partial correlations (pink: positive; green: negative), with deeper color indicating stronger correlations. Values within cells denote partial correlation coefficients, and asterisks indicate statistical significance levels (* *p* < 0.05. ** *p* < 0.01. *** *p* < 0.001). Abbreviations: KCAL, total energy intake (kcal/d). CDI, total Cantonese dietary index score. C1-C8, facets of CDI.

In the GNHS cohort, the Cronbach’s alpha coefficient of the CDI was 0.62 (95% CI: 0.60, 0.64). Spearman correlation coefficients between baseline and the third follow-up CDI scores are presented in Figure 1, ranging from 0.019 (*p* > 0.05) for facet 1 to 0.998 (*p* < 0.001) for facet 7. The intra-class correlation coefficient (ICC) for the total CDI score between baseline and the third follow-up was 0.33 (*p* < 0.001). ICC values for individual facets are presented in Supplementary Table S5.

### 3.4. Validity of the CDI Across GNHS, TCLSIH and NHANES

**Figure 2:**
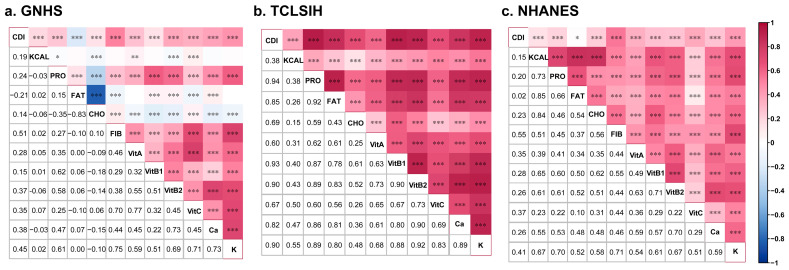
Heatmap of partial Spearman’s correlations between CDI score and nutrients intake across three studies. Partial Spearman’s correlations were adjusted for sex and age. Colors in the heatmap represent the direction and magnitude of partial correlations (pink: positive; blue: negative), with deeper color indicating stronger correlations. Values within cells denote partial correlation coefficients, and asterisks indicate statistical significance levels (* *p* < 0.05. ** *p* < 0.01. *** *p* < 0.001). Abbreviations: CDI, total Cantonese dietary index. KCAL, total energy intake (kcal/d). PRO, protein (g/d). FAT, total fat (g/d). CHO, carbohydrate (g/d). FIB, fiber (g/d). VitA, Vitamin A (ugRE/d). VitB_1_, Vitamin B_1_ (mg/d). VitB_2_, Vitamin B_2_ (mg/d). VitB3, Niacin (mg/d). VitC, Vitamin C (mg/d). VitE, Vitamin E (mg/d). Ca, calcium (mg/d). P, phosphorus (mg/d). K, potassium (mg/d). Na, sodium (mg/d).

**Figure 3:**
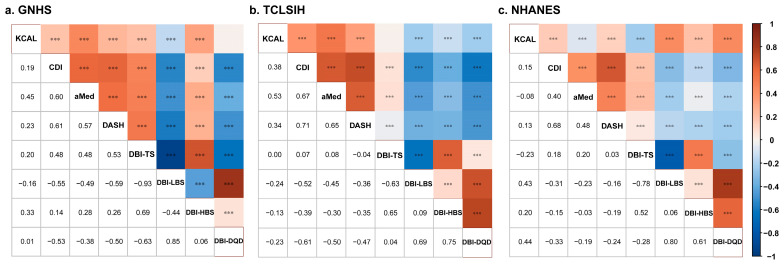
Heatmap of partial Spearman’s correlations between CDI and other diet quality scores across the three studies. Partial Spearman’s correlations were adjusted for sex and age. Colors in the heatmap represent the direction and magnitude of partial correlations (brown: positive; blue: negative), with deeper color indicating stronger correlations. Values within cells denote partial correlation coefficients, and asterisks indicate statistical significance levels (*** *p* < 0.001). Abbreviations: KCAL, total energy intake (kcal/d). CDI, total Cantonese dietary index. aMed, alternative Mediterranean dietary index. DASH, dietary approaches to stop hypertension. DBI, dietary balance index. TS, total score of DBI. LBS, low bound score of DBI. HBS, high bound score of DBI. DQD, dietary quality difference in DBI.

Validity was further evaluated using correlations with nutrient intakes and established diet-quality indices, followed by associations with metabolic syndrome and its components. The correlations between CDI scores and nutrient intakes across the three cohorts are presented in Figure 2. CDI scores were positively correlated with most macro- and micronutrients. The strength of correlations varied across cohorts, ranging from −0.21 (fat) to 0.51 (fiber) in GNHS, from 0.38 (energy) to 0.94 (protein) in TCLSIH, and from 0.02 (fat) to 0.55 (fiber) in NHANES. Partial correlation coefficients between CDI and other diet-quality scores are shown in Figure 3. CDI scores were positively correlated with aMed, DASH, and DBI-TS, and negatively correlated with DBI-LBS and DBI-DQD. The correlations differed across cohorts, ranging from −0.55 (DBI-LBS) to 0.61 (DASH) in GNHS, from −0.61 (DBI-DQD) to 0.71 (DASH) in TCLSIH, and from −0.33 (DBI-DQD) to 0.68 (DASH) in NHANES.

In the GNHS cohort, associations between CDI and prevalent outcomes are presented in Supplementary Table S6. The association between CDI and MetS was linear (all *p* for nonlinearity ≥ 0.262; Supplementary Figure S2). The central finding was that higher CDI scores were inversely associated with metabolic syndrome across the development cohort and two external validation cohorts. After adjustment for covariates (Model 2), each 10-point increase in CDI was associated with a lower prevalence of MetS (OR = 0.78, 95% CI: 0.72, 0.85; *p* = 9.13 × 10^−9^). When analyzed by tertiles, the adjusted OR (95% CI) comparing the highest with the lowest tertile was 0.64 (0.53, 0.76), with a *p* for trend of 9.55 × 10^−7^. Similar associations were observed in the TCLSIH cohort (OR = 0.95, 95% CI: 0.92, 0.98; *p* = 7.31 × 10^−4^) and the NHANES study (OR = 0.84, 95% CI: 0.82, 0.87; *p* = 9.13 × 10^−29^). Associations between CDI and hyperglycemia varied across the three cohorts.

**Figure 4:**
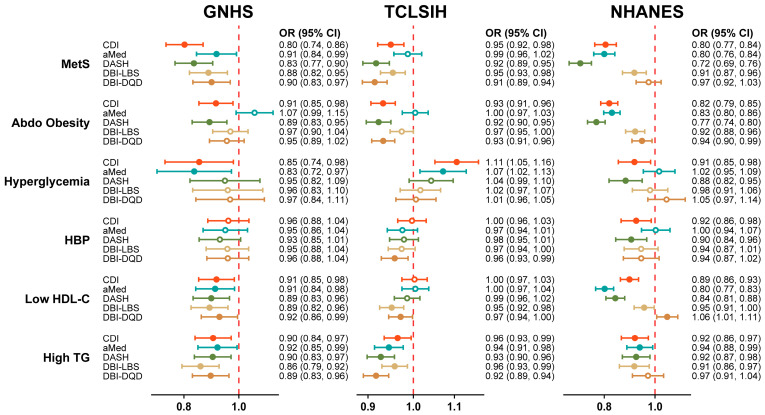
Forest Plot for the association between CDI, aMed, DASH and DBI with MetS and its components in GNHS, TCLSIH, and NHANES studies. The forest plot shows OR and their 95% confidence intervals for different dietary indices with MetS and its components. To facilitate comparisons across indices, negative weight transformations were applied to DQD and LBS, which are positively associated with disease (transformed using exp (1/ln (OR)). Abbreviations: Abdo Obesity, abdominal obesity; aMed, alternative Mediterranean dietary index; CDI, Cantonese dietary index; CI, confidence interval; DASH, dietary approaches to stop hypertension; DBI, dietary balance index; DBI-LBS, low bound score of DBI. DBI-DQD, the dietary quality difference in DBI; GNHS, Guangzhou Nutrition and Health Study; HBP, hypertension; HDL-C, high-density lipoprotein cholesterol; MetS, metabolic syndrome; NHANES, the National Health and Nutrition Examination Survey; OR, odds ratio; TCLSIH, Tianjin Chronic Low-grade Systemic Inflammation and Health; TG, triglycerides.

To ensure comparability across indices with different scoring ranges, all associations are presented per standard deviation increase. Associations between standardized dietary indices and metabolic outcomes are shown in Figure 4, with detailed results provided in Supplementary Table S7.

Associations between CDI and MetS were observed in both the GNHS cohort and external populations with distinct demographic and dietary characteristics. CDI was associated with MetS (GNHS: OR = 0.80, 95% CI: 0.74, 0.86, *p* = 9.13 × 10^−9^; TCLSIH: OR = 0.95, 95% CI: 0.92, 0.98, *p* = 7.31 × 10^−4^; NHANES: OR = 0.80, 95% CI: 0.77, 0.84, *p* = 2.10 × 10^−19^). Associations were also observed for aMed with hyperglycemia (OR = 0.83, 95% CI: 0.72, 0.97; *p* = 1.88 × 10^−2^), DASH with abdominal obesity (OR = 0.89, 95% CI: 0.83, 0.95; *p* = 9.13 × 10^−4^), and DBI-LBS with lipid-related outcomes. In the TCLSIH and NHANES cohorts, DASH was associated with MetS (TCLSIH: OR = 0.92, 95% CI: 0.89, 0.95; *p* = 3.91 × 10^−8^; NHANES: OR = 0.72, 95% CI: 0.69, 0.76; *p* = 2.59 × 10^−39^), and inversely weighted DBI-LBS was associated with low HDL-C in TCLSIH (OR = 0.95, 95% CI: 0.92, 0.98; *p* = 4.20 × 10^−4^). In the NHANES study, CDI was associated with abdominal obesity (OR = 0.82, 95% CI: 0.79, 0.85; *p* = 1.02 × 10^−25^), hypertension (OR = 0.92, 95% CI: 0.86, 0.98; *p* = 1.44 × 10^−2^), and high triglycerides (OR = 0.92, 95% CI: 0.86, 0.97; *p* = 3.50 × 10^−3^). Domain-specific analyses excluding each domain and both the quintile and absolute intake scoring methods yielded broadly similar associations (Supplementary Tables S8 and S9).

## 4. Discussion

In this study, we developed and evaluated the Cantonese Dietary Index (CDI) using data from three independent cohorts, including two Chinese populations and one US population. The CDI demonstrated moderate validity and reliability and was associated with the prevalence of metabolic syndrome across all cohorts. Similar patterns of association were observed in populations with different demographic and dietary characteristics. Compared with existing dietary indices, the CDI showed associations with metabolic outcomes that were comparable. These findings suggest that the CDI may serve as a useful approach for characterizing dietary patterns that integrate both nutritional and culture-specific components.

### 4.1. Development of the CDI Based on Regional Dietary Features

The CDI was developed based on eight key principles of the Cantonese dietary patterns. In addition to common components like vegetables, fruits, and meat, CDI incorporates several region-specific features. In Guangdong, breakfast holds cultural importance within the ‘dim sum’ tradition and is considered the most important meal of the day [41]. Regular consumption of a nutritious breakfast is associated with better health [42,43]. Therefore, breakfast frequency was included to reflect balanced eating behaviors. In addition, tea drinking is also a habitual practice in Cantonese culture. Given the potential health benefits of tea-derived compounds such as polyphenols, theophylline, and caffeine [4,44,45], the CDI adopted an approach similar to the aMed score for alcohol consumption, assigning higher points to regular but moderate tea consumption.

The Cantonese dietary style emphasizes light cooking methods, such as steaming, boiling, and quick stir-frying, while limiting frying and pickling [3,34,46]. Consequently, the frequency of consumption of fried and preserved foods was included in the CDI. Food therapy is another distinctive feature of the Cantonese diet, with frequent consumption of herbal teas (containing ginseng, astragalus, barley, etc.) and long-simmered soups (typically prepared with ingredients like five-fingered peach, Chinese yam, Lycium chinensis, and chenpi) [47,48,49]. These foods are traditionally considered beneficial to health and were therefore included as components of the CDI. Notably, to enhance the feasibility of the CDIs in large-scale studies, these region-specific factors were assessed qualitatively rather than quantitatively.

### 4.2. Performance of the CDI

In this study, the median CDI score was highest in GNHS at 58.5 (52.0, 64.5), followed by TCLSIH at 51.0 (44.5, 57.5) and NHANES at 49.0 (41.0, 57.5). Disparities in food availability and accessibility and dietary habits in different people could explain the difference between the three studies. It is worth noting that all the participants had lower total scores, suggesting that diet quality should be improved in all three populations.

#### 4.2.1. Reliability of the CDI

The CDI reflects overall dietary quality using a composite scoring system, with higher scores indicating healthier dietary patterns. The Cronbach’s alpha coefficient of the CDI is 0.620, which is acceptable compared with other dietary indices developed in different countries, ranging from 0.22 to 0.68 [50,51]. Measurement errors from FFQs, including recall bias and reporting inaccuracy, may attenuate correlations among components and reduce internal consistency. Unlike psychometric scales that are designed to measure a single underlying construct, Cronbach’s alpha has limitations for dietary indices. The CDI captures multiple domains that are not expected to be highly correlated. Thus, moderate internal consistency does not imply poor reliability; rather, it reflects the multidimensional structure of the construct. Greater emphasis was placed on other reliability measures, such as the intra-class correlation coefficient, to assess the long-term stability of the CDI.

The CDI showed moderate stability over 5–6 years with an ICC of 0.332. Given the long interval, this ICC reflects both true dietary changes and measurement variability. Dietary patterns may change due to aging, lifestyle modifications, or health conditions; therefore, moderate ICC is expected and consistent with the dynamic nature of dietary behaviors. Overall, the CDI demonstrated moderate internal consistency and temporal stability.

#### 4.2.2. Validity of the CDI

Compared with other dietary indices, the CDI showed generally stronger associations with metabolic outcomes across populations, with effect sizes comparable to aMed and DASH. Unlike these two indices based on Western dietary patterns, the CDI incorporates both nutritional components and culturally specific behavior. This may explain differences in associations across populations with distinct dietary structures [17,18,52]. Overall, these findings indicate that different dietary indices capture overlapping but not identical aspects of diet quality, and their associations with health outcomes may vary across population contexts and dietary characteristics. Because the study was not designed to formally test the superiority of one dietary index over another, comparisons across indices should be interpreted descriptively and cautiously.

Notably, the CDI showed inconsistent associations with hyperglycemia. This could be explained by several factors. First, hyperglycemia represents a specific metabolic abnormality, whereas the CDI was designed to capture overall dietary quality, which is more strongly related to adiposity, lipid metabolism, and maybe inflammation than with glycemic control alone. In addition, heterogeneity between GNHS and TCLSIH in baseline characteristics, dietary structure, and carbohydrate quality may further contribute to the divergent findings. Moreover, age-related differences and potential reverse causation (diet modification after diagnosis) may bias associations. Finally, the CDI was not specifically designed for glycemic outcomes, which may lead to population-dependent variability. These findings suggest that the CDI should be interpreted primarily as an index of overall Cantonese diet quality and metabolic syndrome-related risk, rather than as a glycemia-specific dietary score.

#### 4.2.3. Potential Biological Mechanisms

Higher CDI scores were consistently associated with lower prevalence of metabolic syndrome across three studies. Although the present study does not allow causal or mechanistic inference due to its observational and predominantly cross-sectional design, several potential biological pathways may explain these associations.

The Cantonese dietary pattern includes higher intake of vegetables, fruits, and aquatic products, providing dietary fiber, polyunsaturated fatty acids, and a range of bioactive compounds. These nutrients are associated with improved lipid profiles and reduced inflammation [53,54]. Moreover, herbal teas and traditional soups also provide polyphenols and other phytochemicals associated with antioxidant and anti-inflammatory effects [47,48,49]. Emerging evidence also suggests that dietary patterns rich in plant-based foods and diverse ingredients may influence the composition and function of the gut microbiota [55,56,57]. Increased intake of fiber and bioactive compounds can promote microbial diversity and the production of short-chain fatty acids [58], which play a role in regulating glucose metabolism, lipid homeostasis, and systemic inflammation [59,60]. In addition, light cooking methods and lower consumption of fried and preserved foods may reduce exposure to pro-inflammatory compounds and excessive sodium intake, both of which are associated with metabolic dysregulation [61]. Together, these mechanisms provide biological plausibility for the observed associations.

### 4.3. Strengths and Limitations

#### 4.3.1. Strengths of the Study

This study is the first to develop a dietary index based on the Cantonese dietary pattern. Several strengths should be noted. First, the CDI was evaluated across three independent cohorts with diverse geographic and demographic backgrounds. Second, large-scale populations with standardized data collection, detailed dietary assessment, and well-defined health outcomes enhanced reliability. Third, the CDI was systematically evaluated for both reliability and validity using multiple approaches. In addition, a wide range of potential confounders was considered in the analyses. Moreover, we assessed whether the associations were primarily driven by dietary intake rather than lifestyle-related behavioral components using domain-specific analyses.

#### 4.3.2. Research Limitations

Several limitations should be considered. First, dietary intake was assessed using self-reported methods, including FFQs in GNHS and TCLSIH and 24-h recalls in NHANES, which are subject to recall bias and measurement error. However, validated dietary assessment tools were used in all cohorts, and averaging repeated measurements helped reduce random error and better reflect habitual intake. In addition, similar associations observed across cohorts using different dietary assessment methods support the robustness of the findings. Second, using a 5–6-year ICC as an indicator of reproducibility has inherent limitations; it primarily reflects long-term stability rather than short-term reproducibility. Over such a time span, ICC values capture both true changes in dietary behaviors and measurement error, making it difficult to disentangle these components. Nevertheless, given that dietary patterns may change with aging, lifestyle, and health status [62], long-term stability also represents an important dimension of reliability in nutritional epidemiology. Moreover, the observational design, with predominantly cross-sectional analyses, limits causal inference. Although we adjusted for a wide range of potential confounders, residual confounding cannot be excluded. However, the consistency of associations across three independent cohorts with different characteristics strengthens the credibility of the findings. Associations between CDI and MetS, as well as its components, should be interpreted as supportive evidence for construct validity and do not imply causality or definitive validation of the CDI. In addition, the application of the CDI in NHANES should be interpreted with caution, as some culturally specific components may not be fully captured. However, this external validation provides insight into whether the CDI reflects general aspects of diet quality beyond its original cultural setting. Finally, sensitivity analyses examined alternative weighting schemes; the CDI was not optimized based on disease outcomes.

### 4.4. Future Perspectives

Because the weighting scheme was based mainly on dietary principles, guidelines, and expert consensus rather than formal data-driven optimization or internal cross-validation, further refinement and validation are warranted in the following aspects: larger and more diverse populations, including prospective cohorts and intervention studies, to better evaluate temporal and potentially causal relationships between Cantonese diet quality and metabolic outcomes; incorporating objective dietary assessment tools and biomarkers of food intake may improve measurement accuracy; and mechanistic studies exploring inflammation, oxidative stress, lipid and glucose metabolism, and the gut microbiota–metabolite axis may provide deeper insight into the biological pathways. In addition, future studies should evaluate the clinical and public health utility of the CDI for risk stratification, dietary intervention assessment, and comparison with established dietary indices across different cultural contexts.

## 5. Conclusions

The CDI was developed to assess adherence to Cantonese dietary patterns. In this study, the CDI showed moderate validity and reliability and was associated with metabolic outcomes across multiple cohorts. These findings suggest that the CDI may be useful for characterizing dietary patterns in the studied populations. Higher CDI scores were associated with a lower prevalence of metabolic syndrome. Taken together, these findings provide preliminary, supportive evidence for the reliability and validity of the CDI, but further prospective, interventional, and clinical validation studies are needed to further validate and refine the CDI across diverse populations. In addition, mechanistic studies exploring pathways such as inflammation and gut microbiota may provide deeper insight into the associations between CDI and metabolic outcomes.

## Figures and Tables

**Table 1:** Scoring Standards of Cantonese Dietary Index.

Facets	Items	Score	Unit	Scoring Criteria				
				1st	2nd	3rd	4th	5th
C1. Wide varieties and balanced ingredients of foods	C11: Dietary variety	0~10		2	4	6	8	10
	C12: Breakfast eating	0~5	d/week	0: <3; 1: 3; 2: 4; 3: 5; 4: 6; 5: 7				
C2. Sufficient vegetables and plentiful fruits.	C21: Total vegetable	1~5	g/1000 kcal	<140	140~180	181~214	215~269	≥270
			Quintiles	1	2	3	4	5
	C22: Dark vegetables	0, 5	Percent, %	0: <50%, 5: ≥50%, dark/total (vegetables)				
	C23: Fruits	2~10	g/1000 kcal	<40	40~69	70~89	90~129	≥130
			Quintiles	2	4	6	8	10
C3. Ample fish and shellfish, moderate meat, poultry, eggs, and dairy products	C31: Total animal foods	1, 3, 5	g/1000 kcal	<80	80~99	100~109	110~129	≥130
			Quintiles	1	3	5	3	1
	C32: Fish and poultry/total animal	1~5	g/1000 kcal	<0.30	0.30~0.34	0.35~0.39	0.40~0.49	≥0.50
			Quintiles	1	2	3	4	5
	C33: Dairy and its products (low-fat)	1~5	g/1000 kcal	<10	10~49	50~139	140~199	≥200
			Quintiles	1	2	3	4	5
C4. Regular consumption of beans, whole grain, nuts, and seeds	C41: Whole grains and non-soybeans *	0~7.5	g/1000 kcal	<2.0	2.0~2.9	3.0~5.9	6.0~9.9	≥10.0
			Quintiles	0	2	4	6	7.5
	C42: Soybeans and nuts *	0~7.5	g/1000 kcal	<5.0	5.0~8.9	9.0~12.9	13.0~19.9	≥20.0
			Quintiles	0	2	4	6	7.5
C5. Fresh ingredients and light cooking style, low sodium and oil	C51: Saturated fatty acids	0~7	g/1000 kcal	<10.0	10.0~11.9	12.0~14.9	15.0~18.9	≥19.0
			Quintiles	7	6	4	2	0
	C52: Salt	0~7	g/1000 kcal	<3.0	3.0~3.4	3.5~3.9	4.0~4.4	≥4.5
			Quintiles	7	6	4	2	0
	C53: Added sugar	0~6	g/1000 kcal	<25	25~34	35~43	44~49	≥50
			Quintiles	6	5	3	1	0
C6. More tea, less alcohol	C61: Tea	0, 2	2: ≥7 times/week, 0: <7 times/week					
	C62: Alcohol	0~4	4: Non-drinker; 3: Former drinker; 2: Current drinker. Deduct 1 point for each instance of intoxication per year, with a minimum score of 0					
C7. Cooking more by steaming, boiling, stewing, and quick stir-frying, less frying, preserving or pickling	C7: Fried and preserved foods	1, 3, 5	5: <1 time/month; 3: 1~3 (time/month) 1: >4 times/week					
C8. Enjoyment of “dimsum” and tea in the morning, frequent consumption of Cantonese-style soup, and paying attention to dietary regimen	C81: Food–medicine homologous substances	0, 2	0: No; 2: Yes					
	C82: Dietary nutritional supplements	0, 2	0: No; 2: Yes					
Total		100						

Note: * dry weight for calculation. Quintiles: population-specific quintiles.

**Table 2:** Characteristics of the study participants in GNHS, TCLSIH, and NHANES ^a^.

Variables	GNHS Cohort *N* = 4025		TCLSIH Cohort *N* = 29,165		NHANES *N* = 28,890	
	Male	Female	Male	Female	Male	Female
Number of participants	1280 (31.8)	2745 (68.2)	16,147 (55.4)	13,018 (44.6)	14,081 (48.7)	14,809 (51.3)
Age, years	60.0 (55.0, 65.0)	57.0 (53.0, 61.0)	44.6 (12.7)	43.1 (12.7)	50.0 (34.0, 64.0)	49.0 (35.0, 64.0)
BMI, kg/m^2^	23.7 (21.7, 25.8)	23.0 (21.0, 25.0)	26.0 (3.5)	23.3 (3.5)	27.8 (24.6, 31.5)	28.4 (24.0, 33.7)
Waist circumference, cm	86.5 (80.7, 91.3)	81.5 (75.5, 88.0)	89.24 (9.46)	76.96 (9.52)	100.0 (90.6, 110.0)	95.6 (85.0, 107.3)
Marital status, %						
Married	1242 (96.7)	2388 (86.7)	14,575 (90.3)	11,322 (87.0)	9288 (66.0)	7955 (53.7)
Others	42 (3.3)	367 (13.3)	1572 (9.7)	1696 (13.0)	4787 (34.0)	6848 (46.3)
Educational attainments, %						
Secondary high school or below	858 (66.8)	2190 79.5)	5640 (34.9)	5027 (38.6)	7031 (49.9)	6882 (46.5)
College degree or above	426 (33.2)	565 (20.5)	10,507 (65.1)	7991 (61.4)	7041 (50.1)	7911 (53.5)
Household monthly income ^b^						
Low income	730 (64.0)	1886 (73.1)	9789 (60.6)	8200 (63.0)	-	-
High income	411 (36.0)	692 (26.8)	6358 (39.4)	4818 (37.0)	-	-
PIR ^c^	-	-	-	-	2.1 (1.2, 4.1)	2.1 (1.1, 3.7)
Smoking, %	654 (50.9)	19 (0.7)	5833 (36.1)	202 (1.6)	7637 (54.3)	5369 (36.3)
Drinking, %	217 (17.0)	63 (2.3)	12,869 (79.7)	5371 (41.3)	11,146 (79.2)	8232 (55.6)
Tea, %	908 (71.0)	1212 (44.2)	13,216 (81.8)	8987 (69.0)	3228 (22.9)	3967 (26.8)
Physical activity, weekly or daily MET-hour ^d^	35.0 (30.0, 46.7)	35.7 (30.6, 50.4)	23.21 (37.03)	19.27 (32.86)	10.0 (6.0, 28.6)	10.0 (4.8, 12.9)
Metabolic syndrome, %	320 (26.1)	828 (31.6)	5371 (33.3)	2177 (16.7)	3511 (30.3)	4314 (36.6)
Abdominal obesity, %	297 (23.2)	712 (25.9)	7969 (49.4)	2665 (20.5)	6057 (44.6)	9746 (68.7)
Hyperglycemia, %	136 (10.9)	203 (7.6)	1662 (10.3)	598 (4.6)	2709 (37.8)	2649 (36.1)
Hypertension, %	567 (44.4)	962 (35.1)	5927 (36.7)	2328 (17.9)	5060 (37.4)	5415 (37.8)
Low HDL-C, %	320 (25.7)	879 (32.8)	4422 (27.4)	3383 (26.0)	3801 (28.3)	4839 (34.4)
High TG, %	391 (31.4)	726 (27.1)	5811 (36.0)	1767 (13.6)	1955 (30.4)	1561 (23.2)

^a^ Data are presented as n (%) for categorical variables and as mean (standard deviation, SD) or median (interquartile range, IQR) for continuous variables, as appropriate. “-” indicates data not available. ^b^ GNHS: Low income, <3000.0 yuan/month. High income, ≥3000.0 yuan/month. TCLSIH: Low income, <10,000 yuan/month. High income, ≥10,000 yuan/month. ^c^ PIR, the ratio of family income to poverty. ^d^ Weekly MET-hours in the TCLSIH cohort, daily MET-hours in the GNHS cohort and the NHANES. Abbreviations: BMI, body mass index; GNHS, Guangzhou Nutrition and Health Study; MET, metabolic equivalent of task; HDL-C, high-density lipoprotein cholesterol; NHANES, the National Health and Nutrition Examination Survey; PIR, the ratio of family income to poverty; TCLSIH, Tianjin Chronic Low-grade Systemic Inflammation and Health; TG, triglycerides.

## Data Availability

The data presented in this study are available on request from the corresponding author due to the data containing information that could compromise participant privacy.

## Supplementary Materials

The following supporting information can be downloaded at: https://www.mdpi.com/article/10.3390/nu18111678/s1; Supplementary Methods: Definitions on Components of CDI, Domain, main role, and rationale of eight core components of the Cantonese dietary pattern, Conversion of FPED food pattern components to gram equivalents, Scoring details of aMED, DASH and DBI, Covariables collection; Figure S1: Flow chart of the GNHS cohort, TCLSIH cohort, and NHANES study; Figure S2: Dose–response associations between CDI and MetS and its components; Table S1: Sensitive analyses of each CDI facet in the GNHS cohort; Table S2: Characteristics of participants in the three studies by tertiles of CDI in different sex; Table S3: The CDI scores of the study participants across three studies; Table S4: Components scores by tertiles between different sex in the GNHS, the TCLSIH, and the NHANES study; Table S5: The intra-class correlation coefficients of the CDI score between the baseline and 3rd follow up in the GNHS cohort; Table S6: Association between CDI and multiple disease in the GNHS cohort, the TCLSIH cohort and NHANES study; Table S7: Association between diseases and diet-quality scores in the GNHS cohort, the TCLSIH cohort and NHANES study; Table S8: Association between diseases and domain scores of CDI across three cohort; Table S9: Association between diseases and two scoring methods of CDI in GNHS and NHANES.

## Abbreviations

The following abbreviations are used in this manuscript:

aMed	Alternative Mediterranean dietary index
Abdo obesity	Abdominal obesity
BMI	Body mass index
CDI	Cantonese dietary index
CI	Confidence interval
DASH	Dietary approaches to stop hypertension
DBI	Dietary balance index
DQD	Diet quality difference
GNHS	Guangzhou Nutrition and Health Study
HBP	Hypertension
HBS	High bound score of dietary balance index
HDL-C	High-density lipoprotein cholesterol
LBS	Low bound score of dietary balance index
MetS	Metabolic syndrome
NHANES	The National Health and Nutrition Examination Survey
OR	Odds ratio
TCLSIH	Tianjin Chronic Low-grade Systemic Inflammation and Health
TG	Triglycerides.
TS	Total score of dietary balance index
