# Supplementary material for: Development and External Validation of the Cantonese Dietary Index: A Population-Based Approach to Assess Diet Quality and Metabolic Risk
Source: Nutrients. 2026 May 24;18(11):1678. doi: 10.3390/nu18111678 (PMC13258514; doi:10.3390/nu18111678)
Supplement: Supplementary file 1 [file nutrients-18-01678-s001.zip › nutrients-4282043-supplementary.pdf]

Contents of Supplemental Materials

Pages	Contents of Supplemental materials
Supplementary Methods	
Page 2-3	Definitions on Components of CDI
Page 4	Domain, main role, and rationale of eight core components of the Cantonese dietary pattern
Page 5	Conversion of FPED food pattern components to gram equivalents
Page 6	Scoring details of aMED, DASH and DBI
Page 7	Covariables collection
Supplementary Figures	
Page 8	Figure S1. Flow chart of the GNHS cohort, TCLSIH cohort, and NHANES study
Page 9	Figure S2. Dose–response associations between CDI and MetS and its components.
Supplementary Tables	
Page 10-12	Table S1. Sensitive analyses of each CDI facet in the GNHS cohort
Page 13-16	Table S2. Characteristics of participants in the three studies by tertiles of CDI in different sex
Page 17	Table S3. The CDI scores of the study participants across three studies
Page 18-22	Table S4. Components scores by tertiles between different sex in the GNHS, the TCLSIH, and the NHANES study
Page 23	Table S5. The intra-class correlation coefficients of the CDI score between the baseline and 3rd follow up in the GNHS cohort
Page 24-26	Table S6. Association between CDI and multiple disease in the GNHS cohort, the TCLSIH cohort and NHANES study
Page 27-29	Table S7. Association between diseases and diet-quality scores in the GNHS cohort, the TCLSIH cohort and NHANES study
Page 30-32	Table S8. Association between diseases and domain scores of CDI across three cohort
Page 33-34	Table S9. Association between diseases and two scoring methods of CDI in GNHS and NHANES

Supplementary Methods

Definitions on Components of CDI

Aspect 1, dietary consumption

1. Vegetables (fresh weight, g/1000 kcal): Various types of fresh vegetables, including root, leafy, gourd, legume, allium, fungi, and algae. Excludes starchy foods (such as potatoes, yams, and tubers) and pickled vegetables (such as sauerkraut, preserved mustard greens).
2. Dark-colored vegetables (fresh weight, g/1000 kcal): Includes dark green, red and yellow vegetables (such as choy sum, mustard greens, broccoli, Chinese cabbage, romaine lettuce, spinach, amaranth, water spinach, tomatoes, peppers, carrots, etc.).
3. Fruits (fresh weight, g/1000 kcal): Various types of fresh fruits, including citrus (oranges, tangerines, grapefruit), stone fruits (peaches, plums), pome fruits (apples, pears), berries (grapes, persimmons), melons (cantaloupe, watermelon), and tropical fruits (mango, papaya, dragon fruit, mangosteen, pineapple, banana, lychee, longan, durian).
4. Total animal foods (fresh weight, g/1000 kcal): Includes red meat [pork (lean/fat, ribs, or skin), lamb, beef (brisket, beef balls)], poultry [chicken, duck, goose (with/without skin)], processed meats and offal (sausages, luncheon meats, liver, brain), freshwater fish (carp, catfish, crucian, perch), saltwater fish (pomfret, grouper, ribbonfish, yellow croaker), mollusks and crustaceans (squid, scallops, oysters, shrimp), and processed aquatic products (small fish with bones, canned fish, salted fish), eggs and egg products.
5. Aquatic Products and poultry (fresh weight, g/1000 kcal):
Aquatic products: Includes freshwater fish (carp, catfish, crucian, perch), saltwater fish (pomfret, grouper, ribbonfish, yellow croaker), mollusks and crustaceans (squid, scallops, oysters, shrimp), and processed aquatic products (small fish with bones, canned fish, salted fish).
Poultry: chicken, duck, goose (with/without skin)
6. Dairy Products (fresh weight, g/1000 kcal): Includes low-fat milk, low-fat milk powder, skim milk, skim milk powder, and yogurt; milk powder and dairy products are converted to liquid milk based on protein content.
7. Whole Grains and Mixed Beans (dry weight, g/1000 kcal):
Whole Grains: Unrefined grains retaining germ, bran, and endosperm, including whole wheat bread, oats, and brown rice.
Mixed Beans: Non-soy legumes such as mung beans, red beans, lentils, peas, and broad beans.
8**. Soybeans and Nuts (dry weight, g/1000 kcal):**
Legumes: Primarily soybeans and their products (including fresh soybeans, tofu varieties, soy milk).
Nuts: Includes peanuts, cashews, walnuts, almonds, pistachios, sesame seeds, etc.
9. Saturated Fat (g/1000 kcal): Total saturated fat intake from the diet.
10. Salt (g/1000 kcal): Average daily salt intake, which can also be estimated using the Tanaka formula based on random urinary sodium levels to calculate 24-hour sodium and salt intake.
11. Added Sugars (g/1000 kcal): Added sugars in foods, including monosaccharides and disaccharides (primarily from sugary beverages, pastries, sweets, desserts, and cooking sugars).
12. Food-Medicine Homologous Substances (g/d): Such as cooling teas (e.g., Five-Finger Peach, Honeysuckle, Banlangen) and slow-cooked soups (e.g., Lotus Lily Soup, Winter Melon Pork Rib Soup, Goji Berry Chicken Soup).

Aspect 2, eating behaviors.

1. Dietary diversity (types/week): Calculated as the total number of food types consumed per week (including grains, tubers, legumes, vegetables, fruits, poultry, meat, fish, eggs, dairy, soy, and nuts).
2. Breakfast Frequency (days/week): Average days per week with breakfast over the past year.
3. Tea (times/week): Average weekly tea-drinking frequency (e.g., green tea, black tea, oolong tea), with each change of tea leaves counted as one instance.
4. Alcohol Consumption:
Consumption status: Whether alcohol is consumed (includes liquor, rice wine, beer, or red wine).
Frequency of drunk (times/year): average times of drunk over the past year.
5. Fried and Preserved Foods Intake Frequency (times/month): Monthly frequency of intake of fried, grilled, and pickled foods (e.g., salted fish, pickled meats, preserved vegetables).
6. Dietary Supplements: Whether dietary supplements were consumed over the past year (e.g., calcium tablets, fish oil, cod liver oil, protein powder).

Domain, main role, and rationale of eight core components of the Cantonese dietary pattern

Facets	Domain	Main role	Rationale
C1. Wide varieties and balanced ingredients of foods	Eating behavior	Both health-related and Cantonese-relevant	Guideline consistency, diverse local food products of Lingnan region
C2. Sufficient vegetables and plentiful fruits.	Dietary consumption	General diet-quality component	Reflect dietary adequacy and guideline consistency
C3. Ample fish and shellfish, moderate meat, poultry, eggs, and dairy products	Dietary consumption	Both health-related and Cantonese-relevant	Reflect Cantonese preference for aquatic foods and healthier animal-food structure
C4. Regular consumption of beans, whole grain, nuts, and seeds	Dietary consumption	General diet-quality component	Reflect dietary adequacy and guideline consistency
C5. Fresh ingredients and light cooking style, low sodium and oil	Dietary consumption	General diet-quality component	Guideline consistency
C6. More tea, less alcohol	Eating behavior	Both health-related and Cantonese-relevant	Reflect Cantonese tea culture and potential bioactive compounds. Guideline consistency for less alcohol
C7. Cooking more by steaming, boiling, stewing, and quick stir-frying, less frying, preserving or pickling	Eating behavior	Both health-related and cultural cooking-style marker	Reflect light cooking and low preserved-food preference
C8. Enjoyment of "dimsum" and tea in the morning, frequent consumption of Cantonese-style soup, and paying attention to dietary regimen (Food–medicine homologous substances)	Eating behavior	Culture-specific marker	Reflect Cantonese dietary-regimen tradition

Conversion of FPED food pattern components to gram equivalents

Food		Average
**Total Fruit** (F_TOTAL)	**Citrus, Melons, and Berries** (F_CITMLB)	165
	**Other Fruits** (F_OTHER)	150
	**Fruit Juice** (F_JUICE)	250
**Total Vegetables** (V_TOTAL)	**Dark Green Vegetables** (V_DRKGR)	80
	**Total Red and Orange Vegetables** (V_REDOR_TOTAL)	170
	**Tomatoes** (V_REDOR_TOMATO)	200
	**Other Red and Orange Vegetables** V_REDOR_OTHER)	145
	**Total Starchy Vegetables** (V_STARCHY_TOTAL)	140
	**Potatoes** (V_STARCHY_POTATO)	135
	**Other Starchy Vegetables** (V_STARCHY_OTHER)	140
	**Other Vegetables** (V_OTHER)	115
	**Beans and Peas (Legumes)** (V_LEGUMES)	60
**Total Grains** (G_TOTAL)	**Whole Grains** (G_WHOLE)	23.86
	**Refined Grains** (G_REFINED)	16
**Total Protein Foods** (PF_ TOTAL)	**Total Meat, Poultry, and Seafood** (PF_MPS_TOTAL)	28.35
	**Meat** (PF_MEAT)	28.35
	**Cured Meat** (PF_CUREDMEAT)	28.35
	**Organ Meat** (PF_ORGAN)	28.35
	**Poultry** (PF_POULT)	28.35
	**Seafood High in n-3 Fatty Acids** (PF_SEAFD_HI)	28.35
	**Seafood Low in n-3 Fatty Acids** (PF_SEAFD_LOW)	28.35
	**Eggs** (PF_EGGS)	50
	**Soy Products** (PF_SOY)	28.35
	**Nuts and Seeds** (PF_NUTSDS)	14.18
	**Beans and Peas (Legumes)** (PF_LEGUMES)	60
**Total Dairy** (D_TOTAL)	**Milk** (D_MILK)	245
	**Yogurt** (D_YOGURT)	245
	**Cheese** (D_CHEESE)	70

Scoring details of aMED, DASH and DBI

- **The alternative Mediterranean dietary score, aMED:** the aMED score has nine items One point each is assigned to those with higher intakes of whole grains, vegetables, fruits, legumes, nuts, and fish, the ratio of monounsaturated to saturated fat, a moderate intake of alcohol, and low intakes of red and processed meats according to the cutoff based upon the sex-specific median in the controls. The total of aMED score ranges from 0 to 9.

- **The Dietary Approaches to Stop Hypertension (DASH) score:** the DASH was based on eight items: high intake of fruits, vegetables, nuts and legumes, low-fat dairy products, and whole grains and low intake of sodium, sweetened beverages, and red and processed meats. The component score for high-intake foods is the population's quintile ranking, as quintile one is assigned 1 point and quintile 5 for 5 points; while for low-intake foods like sodium, the lowest quintile was given a score of 5 points and the highest quintile with 1 point. The total DASH score ranges from 8 to 40.

- **The Dietary Balance Index 16, DBI-16:** the Chinese DBI-16 consists of 8 components, including cereals, vegetables and fruits, dairy and soybeans, animal foods, empty energy food, condiments, diet variety, and drinking water, calculated using different recommendations for food intake according to 11 energy levels by sex. Four scores were calculated under DBI standards: 1) total score (TS) of -72 to 44, representing an average of diet quality; 2) low bound score (LBS), the total of negative scores ranging from 0 to 72, indicating insufficient consumption of food, 3) high bound score (HBS), the total of positive scores ranging from 0 to 44, reflecting excessive intake of foods, and 4) diet quality distance (DQD) by summing the absolute values of each indicator's score, indicating overall dietary imbalance with 0 to 96 points. In this study, LBS and DQD from DBI were employed to assess diet quality.

Covariables collection

Covariates in the GNHS cohort and TCLSIH cohort were collected by face-to-face interviews. The covariates in the GNHS cohort and TCLSIH cohort are listed below:

- **General Information:**

Age: years.
Sex: female and male.
Marital status: married, others (including widowed, divorced, separated, never married, living with partner, or other kinds).
Educational attainments: Secondary high school or below, College degree or above.
Household income per capita: low income (with lower than 3000 yuan in the GNHS), high income (with higher than 3000 yuan in the GNHS).
Smoking: no, yes.
Medication use: **i**ncluding antihyperglycemic agent, antihyperlipidemic drug, and antihypertensive drug use; with the answers of yes or no. Only adjusted in the GNHS cohort and the NHANES study.
Dietary supplement use: no, yes.

- **Physical Activity:**

Physical activity levels over the past month were collected using a standardized questionnaire. The questionnaire divided activities into daily activities (e.g., sleeping, eating, watching TV), work-related activities (e.g., reading, writing, cleaning, standing, or walking in place), and sports or leisure activities (e.g., playing ball games, swimming, running, aerobics, ballroom dancing). Participants reported the type of activity, frequency, and duration for each activity. Physical activity was expressed in METs (Metabolic Equivalent Task), calculated as METs-h/d.

Supplementary Figures

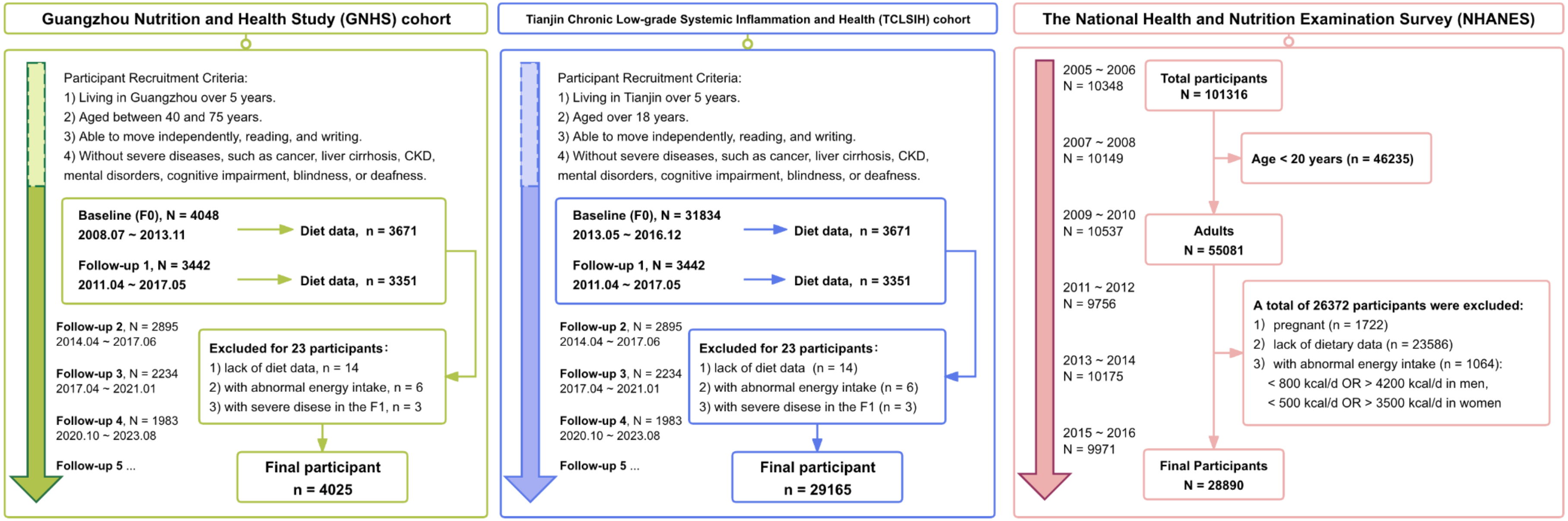

Figure S1. Flow chart of the GNHS cohort, TCLSIH cohort, and NHANES study.

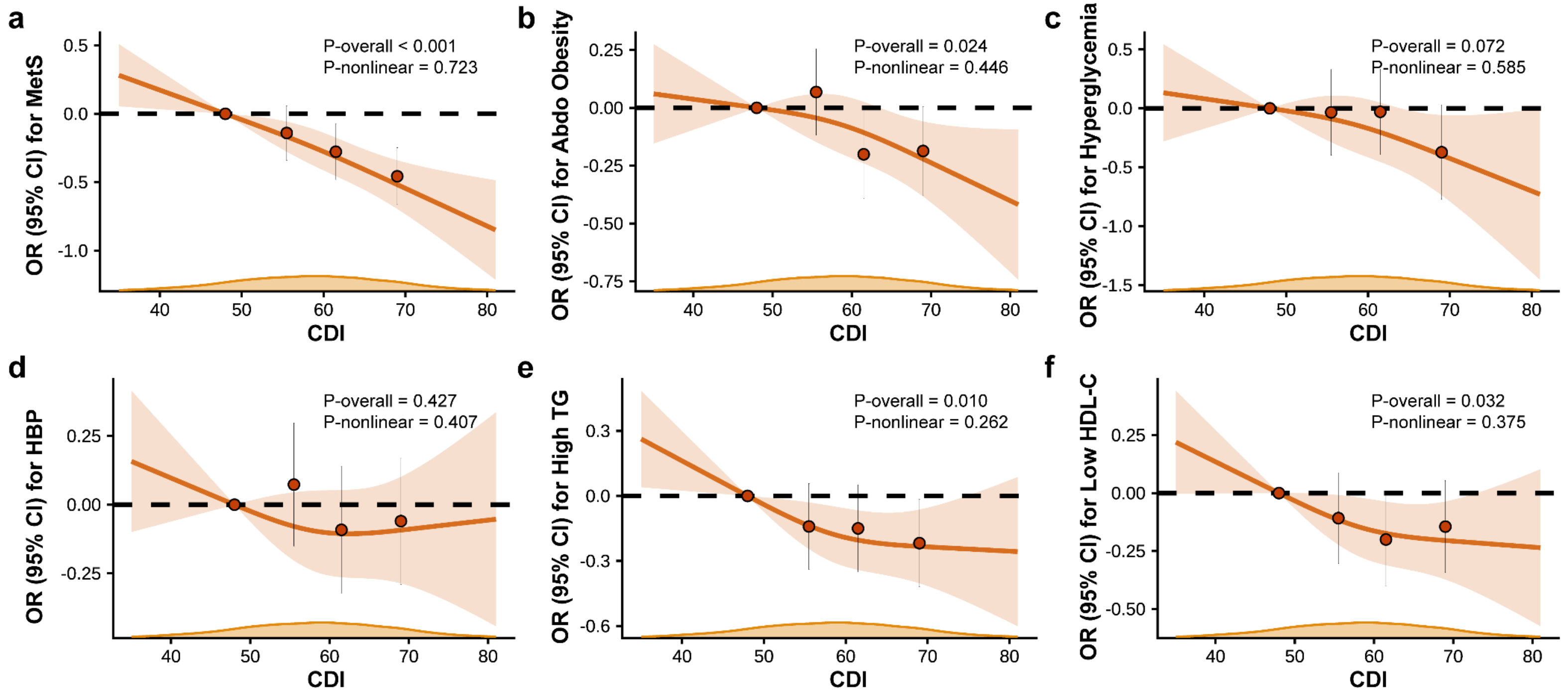

Figure S2. Dose–response associations between CDI and MetS and its components.

Restricted cubic spline analyses illustrating the dose–response relationships between CDI with (a) MetS, (b) abdominal obesity, (c) hyperglycemia, (d) HBP, (e) high TG, and (f) low HDL-C. Solid lines represent adjusted effect estimates, and shaded areas indicate 95% confidence intervals. Circles with error bars denote effect estimates and 95% confidence intervals derived from categorical analyses. The dashed horizontal line represents the reference level (OR = 1). All models were adjusted for age, sex, BMI (unadjusted in models of abdominal obesity and BMI), educational attainments, marital status, smoking status, physical activity, antihypertensive medications, lipid-lowering medications, antidiabetic medications, and total energy intake. The density distribution of the exposure variable is shown at the bottom of each panel.

Supplementary Tables

Table S1. Sensitive analyses of each CDI facet in the GNHS cohort [a]

Weight [b]	Original CDI score				Total CDI score after the facet score × 3				Total CDI score after the facet score × 1/3			
	Model 1		Model 2		Model 1		Model 2		Model 1		Model 2	
	OR (95% CI)	*P*	OR (95% CI)	*P*	OR (95% CI)	*P*	OR (95% CI)	*P*	OR (95% CI)	*P*	OR (95% CI)	*P*
C1												
W1	0.89 (0.83, 0.96)	2.04×10^{-3}	0.90 (0.83, 0.97)	6.06×10^{-3}	0.86 (0.81, 0.93)	3.96×10^{-5}	0.87 (0.81, 0.94)	2.79×10^{-4}	0.94 (0.87, 1.00)	5.03×10^{-2}	0.94 (0.88, 1.01)	7.59×10^{-2}
W2	0.93 (0.87, 0.98)	1.31×10^{-2}	0.95 (0.89, 1.01)	8.96×10^{-2}	0.88 (0.83, 0.94)	1.78×10^{-4}	0.90 (0.85, 0.97)	3.65×10^{-3}	0.95 (0.90, 1.01)	7.45×10^{-2}	0.97 (0.91, 1.03)	2.90×10^{-1}
W3	0.91 (0.85, 0.97)	5.10×10^{-3}	0.93 (0.87, 1.00)	4.40×10^{-2}	0.87 (0.81, 0.93)	5.42×10^{-5}	0.89 (0.83, 0.96)	1.37×10^{-3}	0.94 (0.88, 1.00)	3.96×10^{-2}	0.96 (0.90, 1.02)	1.82×10^{-1}
W4	0.92 (0.86, 0.98)	7.19×10^{-3}	0.94 (0.88, 1.00)	4.85×10^{-2}	0.88 (0.82, 0.94)	9.27×10^{-5}	0.90 (0.84, 0.96)	1.77×10^{-3}	0.94 (0.89, 1.00)	4.72×10^{-2}	0.96 (0.90, 1.02)	1.82×10^{-1}
W5	0.93 (0.87, 0.98)	1.44×10^{-2}	0.95 (0.89, 1.01)	9.93×10^{-2}	0.89 (0.83, 0.94)	1.99×10^{-4}	0.91 (0.85, 0.97)	4.13×10^{-3}	0.95 (0.90, 1.01)	8.09×10^{-2}	0.97 (0.91, 1.03)	3.15×10^{-1}
W6	0.91 (0.85, 0.97)	5.74×10^{-3}	0.93 (0.87, 1.00)	5.00×10^{-2}	0.87 (0.81, 0.93)	6.15×10^{-5}	0.89 (0.83, 0.96)	1.59×10^{-3}	0.94 (0.88, 1.00)	4.38×10^{-2}	0.96 (0.90, 1.02)	2.02×10^{-1}
W7	0.92 (0.86, 0.98)	6.44×10^{-3}	0.93 (0.87, 1.00)	4.29×10^{-2}	0.88 (0.82, 0.94)	8.21×10^{-5}	0.89 (0.83, 0.96)	1.54×10^{-3}	0.94 (0.89, 1.00)	4.29×10^{-2}	0.96 (0.90, 1.02)	1.65×10^{-1}
C2												
W1	0.89 (0.83, 0.96)	2.04×10^{-3}	0.90 (0.83, 0.97)	6.06×10^{-3}	0.93 (0.88, 0.99)	1.37×10^{-2}	0.94 (0.89, 1.00)	4.61×10^{-2}	0.88 (0.82, 0.95)	1.48×10^{-3}	0.89 (0.82, 0.96)	3.14×10^{-3}
W2	0.93 (0.87, 0.98)	1.31×10^{-2}	0.95 (0.89, 1.01)	8.96×10^{-2}	0.98 (0.94, 1.02)	3.06×10^{-1}	1.00 (0.95, 1.04)	8.53×10^{-1}	0.88 (0.82, 0.95)	3.82×10^{-4}	0.90 (0.83, 0.96)	3.33×10^{-3}
W3	0.91 (0.85, 0.97)	5.10×10^{-3}	0.93 (0.87, 1.00)	4.40×10^{-2}	0.97 (0.93, 1.02)	2.66×10^{-1}	0.99 (0.94, 1.05)	7.94×10^{-1}	0.87 (0.81, 0.93)	1.27×10^{-4}	0.88 (0.82, 0.95)	1.36×10^{-3}
W4	0.92 (0.86, 0.98)	7.19×10^{-3}	0.94 (0.88, 1.00)	4.85×10^{-2}	0.97 (0.93, 1.02)	2.66×10^{-1}	0.99 (0.94, 1.04)	7.68×10^{-1}	0.88 (0.83, 0.94)	3.26×10^{-4}	0.90 (0.83, 0.96)	2.31×10^{-3}
W5	0.93 (0.87, 0.98)	1.44×10^{-2}	0.95 (0.89, 1.01)	9.93×10^{-2}	0.98 (0.94, 1.02)	3.14×10^{-1}	1.00 (0.95, 1.04)	8.72×10^{-1}	0.88 (0.82, 0.95)	4.48×10^{-4}	0.90 (0.84, 0.97)	4.07×10^{-3}
W6	0.91 (0.85, 0.97)	5.74×10^{-3}	0.93 (0.87, 1.00)	5.00×10^{-2}	0.97 (0.93, 1.02)	2.76×10^{-1}	0.99 (0.94, 1.05)	8.16×10^{-1}	0.87 (0.81, 0.93)	1.50×10^{-4}	0.88 (0.82, 0.96)	1.68×10^{-3}
W7	0.92 (0.86, 0.98)	6.44×10^{-3}	0.93 (0.87, 1.00)	4.29×10^{-2}	0.97 (0.93, 1.02)	2.57×10^{-1}	0.99 (0.94, 1.04)	7.46×10^{-1}	0.88 (0.82, 0.94)	2.78×10^{-4}	0.89 (0.83, 0.96)	1.88×10^{-3}
C3												
W1	0.89 (0.83, 0.96)	2.04×10^{-3}	0.90 (0.83, 0.97)	6.06×10^{-3}	0.91 (0.85, 0.97)	3.20×10^{-3}	0.91 (0.85, 0.97)	6.45×10^{-3}	0.89 (0.83, 0.96)	2.43×10^{-3}	0.90 (0.84, 0.97)	8.02×10^{-3}
W2	0.93 (0.87, 0.98)	1.31×10^{-2}	0.95 (0.89, 1.01)	8.96×10^{-2}	0.92 (0.87, 0.97)	2.60×10^{-3}	0.93 (0.88, 0.98)	1.14×10^{-2}	0.94 (0.89, 1.00)	4.63×10^{-2}	0.97 (0.91, 1.03)	2.78×10^{-1}
W3	0.91 (0.85, 0.97)	5.10×10^{-3}	0.93 (0.87, 1.00)	4.40×10^{-2}	0.91 (0.86, 0.96)	1.37×10^{-3}	0.92 (0.87, 0.98)	6.09×10^{-3}	0.92 (0.86, 0.99)	2.05×10^{-2}	0.95 (0.89, 1.02)	1.66×10^{-1}
W4	0.92 (0.86, 0.98)	7.19×10^{-3}	0.94 (0.88, 1.00)	4.85×10^{-2}	0.92 (0.87, 0.97)	2.01×10^{-3}	0.93 (0.87, 0.98)	6.61×10^{-3}	0.93 (0.87, 0.99)	3.38×10^{-2}	0.96 (0.90, 1.03)	2.19×10^{-1}
W5	0.93 (0.87, 0.98)	1.44×10^{-2}	0.95 (0.89, 1.01)	9.93×10^{-2}	0.92 (0.87, 0.97)	2.83×10^{-3}	0.93 (0.88, 0.98)	1.26×10^{-2}	0.94 (0.89, 1.00)	5.07×10^{-2}	0.97 (0.91, 1.03)	3.03×10^{-1}

Weight [b]	Original CDI score				Total CDI score after the facet score × 3				Total CDI score after the facet score × 1/3			
	Model 1		Model 2		Model 1		Model 2		Model 1		Model 2	
	OR (95% CI)	*P*	OR (95% CI)	*P*	OR (95% CI)	*P*	OR (95% CI)	*P*	OR (95% CI)	*P*	OR (95% CI)	*P*
W6	0.91 (0.85, 0.97)	5.74×10^{-3}	0.93 (0.87, 1.00)	5.00×10^{-2}	0.91 (0.86, 0.96)	1.51×10^{-3}	0.92 (0.87, 0.98)	6.79×10^{-3}	0.93 (0.87, 0.99)	2.29×10^{-2}	0.95 (0.89, 1.02)	1.86×10^{-1}
W7	0.92 (0.86, 0.98)	6.44×10^{-3}	0.93 (0.87, 1.00)	4.29×10^{-2}	0.92 (0.87, 0.97)	1.86×10^{-3}	0.92 (0.87, 0.98)	6.01×10^{-3}	0.93 (0.87, 0.99)	3.04×10^{-2}	0.96 (0.89, 1.02)	1.97×10^{-1}
C4												
W1	0.89 (0.83, 0.96)	2.04×10^{-3}	0.90 (0.83, 0.97)	6.06×10^{-3}	0.87 (0.82, 0.94)	1.27×10^{-4}	0.89 (0.82, 0.95)	8.46×10^{-4}	0.90 (0.84, 0.97)	6.15×10^{-3}	0.91 (0.84, 0.98)	1.36×10^{-2}
W2	0.93 (0.87, 0.98)	1.31×10^{-2}	0.95 (0.89, 1.01)	8.96×10^{-2}	0.91 (0.86, 0.96)	8.39×10^{-4}	0.93 (0.88, 0.99)	1.79×10^{-2}	0.94 (0.88, 1.00)	4.54×10^{-2}	0.96 (0.90, 1.02)	1.83×10^{-1}
W3	0.91 (0.85, 0.97)	5.10×10^{-3}	0.93 (0.87, 1.00)	4.40×10^{-2}	0.89 (0.84, 0.95)	3.08×10^{-4}	0.92 (0.86, 0.98)	8.48×10^{-3}	0.93 (0.87, 0.99)	2.17×10^{-2}	0.94 (0.88, 1.01)	1.02×10^{-1}
W4	0.92 (0.86, 0.98)	7.19×10^{-3}	0.94 (0.88, 1.00)	4.85×10^{-2}	0.90 (0.85, 0.95)	4.68×10^{-4}	0.92 (0.87, 0.98)	9.81×10^{-3}	0.93 (0.87, 0.99)	2.74×10^{-2}	0.95 (0.89, 1.01)	1.06×10^{-1}
W5	0.93 (0.87, 0.98)	1.44×10^{-2}	0.95 (0.89, 1.01)	9.93×10^{-2}	0.91 (0.86, 0.96)	9.34×10^{-4}	0.93 (0.88, 0.99)	2.00×10^{-2}	0.94 (0.89, 1.00)	4.96×10^{-2}	0.96 (0.90, 1.02)	2.01×10^{-1}
W6	0.91 (0.85, 0.97)	5.74×10^{-3}	0.93 (0.87, 1.00)	5.00×10^{-2}	0.90 (0.84, 0.95)	3.48×10^{-4}	0.92 (0.86, 0.98)	9.64×10^{-3}	0.93 (0.87, 0.99)	2.41×10^{-2}	0.95 (0.88, 1.01)	1.15×10^{-1}
W7	0.92 (0.86, 0.98)	6.44×10^{-3}	0.93 (0.87, 1.00)	4.29×10^{-2}	0.90 (0.85, 0.95)	4.16×10^{-4}	0.92 (0.87, 0.98)	8.66×10^{-3}	0.93 (0.87, 0.99)	2.48×10^{-2}	0.95 (0.88, 1.01)	9.52×10^{-2}
C5												
W1	0.89 (0.83, 0.96)	2.04×10^{-3}	0.90 (0.83, 0.97)	6.06×10^{-3}	0.90 (0.83, 0.97)	6.85×10^{-3}	0.90 (0.83, 0.98)	1.50×10^{-2}	0.90 (0.84, 0.96)	1.81×10^{-3}	0.91 (0.85, 0.97)	5.65×10^{-3}
W2	0.93 (0.87, 0.98)	1.31×10^{-2}	0.95 (0.89, 1.01)	8.96×10^{-2}	0.90 (0.84, 0.96)	1.63×10^{-3}	0.92 (0.86, 0.99)	2.41×10^{-2}	0.94 (0.88, 0.99)	2.58×10^{-2}	0.96 (0.90, 1.01)	1.36×10^{-1}
W3	0.91 (0.85, 0.97)	5.10×10^{-3}	0.93 (0.87, 1.00)	4.40×10^{-2}	0.87 (0.80, 0.93)	1.95×10^{-4}	0.89 (0.83, 0.97)	5.21×10^{-3}	0.93 (0.87, 0.99)	1.68×10^{-2}	0.95 (0.89, 1.01)	9.36×10^{-2}
W4	0.92 (0.86, 0.98)	7.19×10^{-3}	0.94 (0.88, 1.00)	4.85×10^{-2}	0.88 (0.82, 0.95)	7.40×10^{-4}	0.91 (0.84, 0.98)	1.10×10^{-2}	0.93 (0.87, 0.99)	1.54×10^{-2}	0.95 (0.89, 1.01)	7.90×10^{-2}
W5	0.93 (0.87, 0.98)	1.44×10^{-2}	0.95 (0.89, 1.01)	9.93×10^{-2}	0.90 (0.84, 0.96)	1.82×10^{-3}	0.92 (0.86, 0.99)	2.72×10^{-2}	0.94 (0.88, 0.99)	2.83×10^{-2}	0.96 (0.90, 1.02)	1.50×10^{-1}
W6	0.91 (0.85, 0.97)	5.74×10^{-3}	0.93 (0.87, 1.00)	5.00×10^{-2}	0.87 (0.80, 0.94)	2.22×10^{-4}	0.90 (0.83, 0.97)	6.04×10^{-3}	0.93 (0.87, 0.99)	1.87×10^{-2}	0.95 (0.89, 1.01)	1.05×10^{-1}
W7	0.92 (0.86, 0.98)	6.44×10^{-3}	0.93 (0.87, 1.00)	4.29×10^{-2}	0.88 (0.82, 0.95)	6.53×10^{-4}	0.91 (0.84, 0.98)	9.55×10^{-3}	0.93 (0.87, 0.98)	1.38×10^{-2}	0.94 (0.89, 1.00)	7.06×10^{-2}
C6												
W1	0.89 (0.83, 0.96)	2.04×10^{-3}	0.90 (0.83, 0.97)	6.06×10^{-3}	0.96 (0.90, 1.02)	2.07×10^{-1}	0.96 (0.90, 1.02)	1.67×10^{-1}	0.87 (0.81, 0.94)	1.34×10^{-4}	0.89 (0.82, 0.95)	1.14×10^{-3}
W2	0.93 (0.87, 0.98)	1.31×10^{-2}	0.95 (0.89, 1.01)	8.96×10^{-2}	0.98 (0.92, 1.04)	5.43×10^{-1}	0.99 (0.93, 1.06)	7.86×10^{-1}	0.91 (0.86, 0.97)	1.65×10^{-3}	0.93 (0.88, 0.99)	2.64×10^{-2}
W3	0.91 (0.85, 0.97)	5.10×10^{-3}	0.93 (0.87, 1.00)	4.40×10^{-2}	0.98 (0.91, 1.04)	4.86×10^{-1}	0.99 (0.92, 1.06)	6.90×10^{-1}	0.89 (0.84, 0.95)	3.77×10^{-4}	0.92 (0.86, 0.98)	9.03×10^{-3}
W4	0.92 (0.86, 0.98)	7.19×10^{-3}	0.94 (0.88, 1.00)	4.85×10^{-2}	0.98 (0.92, 1.04)	5.12×10^{-1}	0.99 (0.92, 1.06)	7.15×10^{-1}	0.90 (0.85, 0.96)	6.54×10^{-4}	0.92 (0.87, 0.98)	1.05×10^{-2}
W5	0.93 (0.87, 0.98)	1.44×10^{-2}	0.95 (0.89, 1.01)	9.93×10^{-2}	0.98 (0.92, 1.05)	6.02×10^{-1}	0.99 (0.93, 1.06)	8.82×10^{-1}	0.91 (0.86, 0.97)	1.73×10^{-3}	0.93 (0.88, 0.99)	2.78×10^{-2}
W6	0.91 (0.85, 0.97)	5.74×10^{-3}	0.93 (0.87, 1.00)	5.00×10^{-2}	0.98 (0.92, 1.05)	5.44×10^{-1}	0.99 (0.92, 1.06)	7.86×10^{-1}	0.89 (0.84, 0.95)	4.01×10^{-4}	0.92 (0.86, 0.98)	9.62×10^{-3}
W7	0.92 (0.86, 0.98)	6.44×10^{-3}	0.93 (0.87, 1.00)	4.29×10^{-2}	0.98 (0.92, 1.04)	4.58×10^{-1}	0.98 (0.92, 1.05)	6.27×10^{-1}	0.90 (0.85, 0.96)	6.17×10^{-4}	0.92 (0.86, 0.98)	9.88×10^{-3}

Weight [b]	Original CDI score				Total CDI score after the facet score × 3				Total CDI score after the facet score × 1/3			
	Model 1		Model 2		Model 1		Model 2		Model 1		Model 2	
	OR (95% CI)	*P*	OR (95% CI)	*P*	OR (95% CI)	*P*	OR (95% CI)	*P*	OR (95% CI)	*P*	OR (95% CI)	*P*
C7												
W1	0.89 (0.83, 0.96)	2.04×10^{-3}	0.90 (0.83, 0.97)	6.06×10^{-3}	0.90 (0.84, 0.97)	6.53×10^{-3}	0.91 (0.84, 0.98)	1.61×10^{-2}	0.89 (0.83, 0.96)	1.47×10^{-3}	0.90 (0.83, 0.97)	4.59×10^{-3}
W2	0.93 (0.87, 0.98)	1.31×10^{-2}	0.95 (0.89, 1.01)	8.96×10^{-2}	0.93 (0.87, 0.99)	2.03×10^{-2}	0.95 (0.89, 1.01)	1.23×10^{-1}	0.93 (0.87, 0.98)	1.15×10^{-2}	0.95 (0.89, 1.01)	8.12×10^{-2}
W3	0.91 (0.85, 0.97)	5.10×10^{-3}	0.93 (0.87, 1.00)	4.40×10^{-2}	0.91 (0.85, 0.98)	8.88×10^{-3}	0.94 (0.87, 1.00)	6.59×10^{-2}	0.91 (0.85, 0.97)	4.34×10^{-3}	0.93 (0.87, 1.00)	3.89×10^{-2}
W4	0.92 (0.86, 0.98)	7.19×10^{-3}	0.94 (0.88, 1.00)	4.85×10^{-2}	0.92 (0.86, 0.98)	1.19×10^{-2}	0.94 (0.88, 1.01)	7.04×10^{-2}	0.92 (0.86, 0.98)	6.20×10^{-3}	0.93 (0.87, 1.00)	4.32×10^{-2}
W5	0.93 (0.87, 0.98)	1.44×10^{-2}	0.95 (0.89, 1.01)	9.93×10^{-2}	0.93 (0.87, 0.99)	2.23×10^{-2}	0.95 (0.89, 1.02)	1.36×10^{-1}	0.93 (0.87, 0.98)	1.26×10^{-2}	0.95 (0.89, 1.01)	9.01×10^{-2}
W6	0.91 (0.85, 0.97)	5.74×10^{-3}	0.93 (0.87, 1.00)	5.00×10^{-2}	0.91 (0.85, 0.98)	9.95×10^{-3}	0.94 (0.87, 1.01)	7.43×10^{-2}	0.91 (0.85, 0.97)	4.88×10^{-3}	0.93 (0.87, 1.00)	4.42×10^{-2}
W7	0.92 (0.86, 0.98)	6.44×10^{-3}	0.93 (0.87, 1.00)	4.29×10^{-2}	0.92 (0.86, 0.98)	1.07×10^{-2}	0.94 (0.88, 1.00)	6.27×10^{-2}	0.92 (0.86, 0.97)	5.54×10^{-3}	0.93 (0.87, 1.00)	3.82×10^{-2}
C8												
W1	0.89 (0.83, 0.96)	2.04×10^{-3}	0.90 (0.83, 0.97)	6.06×10^{-3}	0.89 (0.83, 0.96)	2.17×10^{-3}	0.90 (0.84, 0.97)	7.79×10^{-3}	0.89 (0.83, 0.96)	2.09×10^{-3}	0.90 (0.84, 0.97)	5.75×10^{-3}
W2	0.93 (0.87, 0.98)	1.31×10^{-2}	0.95 (0.89, 1.01)	8.96×10^{-2}	0.93 (0.87, 0.99)	1.73×10^{-2}	0.95 (0.89, 1.01)	1.16×10^{-1}	0.93 (0.87, 0.98)	1.20×10^{-2}	0.95 (0.89, 1.01)	8.23×10^{-2}
W3	0.91 (0.85, 0.97)	5.10×10^{-3}	0.93 (0.87, 1.00)	4.40×10^{-2}	0.91 (0.85, 0.98)	7.25×10^{-3}	0.93 (0.87, 1.00)	6.12×10^{-2}	0.91 (0.85, 0.97)	4.59×10^{-3}	0.93 (0.87, 1.00)	3.95×10^{-2}
W4	0.92 (0.86, 0.98)	7.19×10^{-3}	0.94 (0.88, 1.00)	4.85×10^{-2}	0.92 (0.86, 0.98)	9.87×10^{-3}	0.94 (0.88, 1.00)	6.58×10^{-2}	0.92 (0.86, 0.98)	6.53×10^{-3}	0.93 (0.87, 1.00)	4.39×10^{-2}
W5	0.93 (0.87, 0.98)	1.44×10^{-2}	0.95 (0.89, 1.01)	9.93×10^{-2}	0.93 (0.87, 0.99)	1.90×10^{-2}	0.95 (0.89, 1.01)	1.28×10^{-1}	0.93 (0.87, 0.98)	1.32×10^{-2}	0.95 (0.89, 1.01)	9.14×10^{-2}
W6	0.91 (0.85, 0.97)	5.74×10^{-3}	0.93 (0.87, 1.00)	5.00×10^{-2}	0.91 (0.85, 0.98)	8.12×10^{-3}	0.94 (0.87, 1.01)	6.90×10^{-2}	0.91 (0.85, 0.97)	5.16×10^{-3}	0.93 (0.87, 1.00)	4.50×10^{-2}
W7	0.92 (0.86, 0.98)	6.44×10^{-3}	0.93 (0.87, 1.00)	4.29×10^{-2}	0.92 (0.86, 0.98)	8.86×10^{-3}	0.94 (0.88, 1.00)	5.86×10^{-2}	0.92 (0.86, 0.97)	5.84×10^{-3}	0.93 (0.87, 1.00)	3.87×10^{-2}

Note: Model 1, adjusted for age, and sex. Model 2, adjusted for age, sex, educational attainments, marital status, smoking status, physical activity, antihypertensive medications, lipid-lowering medications, antidiabetic medications, and total energy intake.

[a] Data are presented by ORs (95% CI) and P values.

[b] W: predefined weighted system of the CDI score.

Abbreviations: CDI, Cantonese dietary index. C1-C8, facet 1-8 of the CDI. GNHS, Guangzhou Nutrition and Health Study.

Table S2. Characteristics of participants in the three studies by tertiles of CDI in different sex [a]

Characteristic	The CDI scores							
	Male				Female			
	Tertile 1	Tertile 2	Tertile 3	*P*	Tertile 1	Tertile 2	Tertile 3	*P*
GNHS								
**Number of Participants**	448	415	417		959	891	895	
Range (min, max)	30.0, 53.0	53.1, 61.0	61.1, 84.5		27.0, 55.0	55.1, 63.0	63.1, 84.5	
**Age**	59.0 (55.0, 64.1)	59.8 (55.0, 65.0)	60.0 (56.0, 66.0)	0.129	56.0 (53.0, 61.0)	57.0 (53.0, 61.0)	57.0 (53.0, 61.7)	0.449
**Marital status, %**				0.240				0.082
Married	427 (95.3)	403 (97.1)	408 (97.8)		809 (84.4)	781 (87.7)	788 (88.0)	
Others	21 (4.7)	12 (2.9)	9 (2.2)		150 (15.6)	110 (12.3)	107 (12.0)	
**Educational attainments, %**				0.018				0.016
Secondary high school or below	323 (72.1)	283 (68.2)	248 (59.5)		779 (81.2)	715 (80.2)	687 (76.8)	
College degree or above	125 (27.9)	132 (31.8)	169 (40.5)		180 (18.8)	176 (19.8)	208 (23.2)	
**Household monthly income** [b]				0.001				0.002
Low income	288 (73.3)	229 (61.2)	213 (57.0)		703 (77.8)	601 (72.6)	581 (68.8)	
High income	105 (26.7)	145 (38.8)	161 (43.0)		201 (22.2)	227 (27.4)	264 (31.2)	
**Smoking, %**	256 (57.1)	207 (49.9)	191 (45.8)	0.022	10 (1.0)	5 (0.6)	4 (0.4)	0.365
**Drinking, %**	84 (18.8)	71 (17.1)	62 (14.9)	0.591	19 (2.0)	21 (2.4)	23 (2.6)	0.738
**Tea, %**	298 (66.5)	292 (70.5)	318 (76.4)	0.03	395 (41.2)	381 (42.9)	434 (48.7)	0.013
**Physical activity, weekly or daily MET-hour** [d]	34.1 (29.6, 49.9)	34.9 (30.3, 46.8)	35.9 (30.5, 45.2)	0.669	35.7 (30.0, 56.5)	35.3 (30.3, 53.8)	36.1 (31.2, 45.4)	0.816
**BMI, kg/m^2**	23.7 (21.7, 25.7)	23.7 (21.6, 25.7)	23.8 (21.7, 25.9)	0.813	23.1 (21.2, 25.4)	22.9 (21.1, 25.1)	22.7 (20.7, 24.6)	0.010
**Waist Circumference, cm**	86.5 (80.5, 91.5)	86.0 (80.5, 91.0)	86.5 (81.5, 91.4)	0.813	82.0 (76.0, 88.5)	81.5 (76.0, 88.1)	81.0 (74.9, 87.0)	0.010
**Metabolic syndrome, %**	129 (30.5)	92 (23.1)	99 (24.4)	0.129	323 (35.8)	279 (32.5)	226 (26.4)	0.001

Characteristic	The CDI scores							
	Male				Female			
	Tertile 1	Tertile 2	Tertile 3	*P*	Tertile 1	Tertile 2	Tertile 3	*P*
Abdominal obesity, %	107 (23.9)	91 (22.0)	98 (23.6)	0.813	270 (28.2)	241 (27.1)	200 (22.4)	0.033
Hyperglycemia, %	47 (10.9)	48 (11.9)	41 (10.0)	0.813	80 (8.6)	70 (8.0)	53 (6.0)	0.176
Hypertension, %	192 (42.9)	189 (45.7)	186 (44.7)	0.813	347 (36.3)	322 (36.2)	293 (32.9)	0.339
High TG, %	149 (34.5)	114 (28.4)	128 (31.1)	0.362	270 (29.1)	240 (27.5)	216 (24.6)	0.176
Low HDL-C, %	140 (32.4)	84 (20.9)	96 (23.4)	0.004	314 (33.8)	290 (33.2)	275 (31.4)	0.611
TCLSIH								
Number of Participants	5665	5101	5381		4392	4444	4182	
Range (min, max)	15.0, 47.0	47.5, 55.5	26.0, 85.0		15.0, 49.0	49.5, 58.0	58.5, 83.0	
Age	41.3 (33.3, 50.0)	43.4 (34.0, 52.7)	45.9 (35.4, 57.6)	< 0.001	38.8 (31.7, 47.5)	41.1 (33.1, 51.3)	44.5 (34.2, 56.3)	< 0.001
Marital status, %				< 0.001				< 0.001
Married	660 (11.6)	475 (9.3)	437 (8.1)		716 (16.3)	570 (12.8)	410 (9.8)	
Others	5005 (88.4)	4626 (90.7)	4944 (91.9)		3676 (83.7)	3874 (87.2)	3772 (90.2)	
Educational attainments, %				0.014				< 0.001
Secondary high school or below	2040 (36.0)	1703 (33.4)	1897 (35.2)		1640 (37.3)	1662 (37.4)	1725 (41.2)	
College degree or above	3625 (64.0)	3398 (66.6)	3484 (64.8)		2752 (62.7)	2782 (62.6)	2457 (58.8)	
Household monthly income [b]				0.025				0.594
Low income	3483 (61.5)	3123 (61.2)	3183 (59.2)		2776 (63.2)	2773 (62.4)	2651 (63.4)	
High income	2182 (38.5)	1978 (38.8)	2198 (40.8)		1616 (36.8)	1671 (37.6)	1531 (36.6)	
Smoking, %	2463 (43.5)	1837 (36.0)	1533 (28.5)	< 0.001	95 (2.2)	70 (1.6)	37 (0.9)	< 0.001
Drinking, %	4668 (82.4)	4158 (81.5)	4043 (75.1)	< 0.001	2050 (46.7)	1842 (41.4)	1479 (35.4)	< 0.001
Tea, %	4383 (77.4)	4254 (83.4)	4579 (85.1)	< 0.001	2833 (64.5)	3132 (70.5)	3022 (72.3)	< 0.001

Characteristic	The CDI scores							
	Male				Female			
	Tertile 1	Tertile 2	Tertile 3	*P*	Tertile 1	Tertile 2	Tertile 3	*P*
Physical activity, weekly or daily MET-hour [d]	11.0 (3.1, 23.1)	12.6 (4.9, 27.6)	17.1 (7.7, 33.3)	< 0.001	8.2 (1.6, 19.2)	11.0 (3.7, 23.1)	13.0 (5.5, 26.2)	< 0.001
BMI, kg/m^2	25.8 (23.6, 28.2)	25.8 (23.6, 28.1)	25.8 (23.7, 28.0)	0.987	22.6 (20.6, 25.2)	23.0 (20.9, 25.3)	23.2 (21.0, 25.5)	< 0.001
Waist Circumference, cm	89.0 (83.0, 96.0)	89.0 (83.0, 95.0)	89.0 (83.0, 95.0)	0.462	75.0 (69.0, 82.0)	76.0 (70.0, 83.0)	77.0 (70.0, 84.0)	< 0.001
Metabolic syndrome, %	1900 (33.5)	1694 (33.2)	1777 (33.0)	0.8435	639 (14.6)	719 (16.2)	819 (19.6)	< 0.001
Abdominal obesity, %	2815 (49.7)	2532 (49.6)	2622 (48.7)	0.531	838 (19.1)	888 (20.0)	939 (22.4)	< 0.001
Hyperglycemia, %	492 (8.7)	498 (9.8)	672 (12.5)	< 0.001	152 (3.5)	185 (4.2)	261 (6.2)	< 0.001
Hypertension, %	1925 (34.0)	1867 (36.6)	2135 (39.7)	< 0.001	622 (14.2)	748 (16.8)	958 (22.9)	< 0.001
High TG, %	2132 (37.6)	1860 (36.5)	1819 (33.8)	< 0.001	515 (11.7)	600 (13.5)	652 (15.6)	< 0.001
Low HDL-C, %	1635 (28.9)	1364 (26.7)	1423 (26.4)	0.008	1106 (25.2)	1160 (26.1)	1117 (26.7)	0.266
NHANES								
Number of Participants	4754	4769	4558		5230	4787	4792	
Range (min, max)	14.0, 43.0	43.1, 53.5	53.6, 89.0		16.0, 45.0	45.1, 55.5	55.6, 89.0	
Age, year	45.0 (31.0, 61.0)	49.0 (35.0, 64.0)	54.0 (39.0, 68.0)	<0.001	44.0 (31.0, 59.0)	51.0 (36.0, 65.0)	54.0 (40.0, 67.0)	<0.001
PIR [c]	2.1 (1.1, 3.4)	2.1 (1.2, 3.9)	2.6 (1.6, 5.0)	<0.001	1.8 (1.0, 3.2)	2.1 (1.1, 3.6)	2.2 (1.4, 4.4)	<0.001
Marital status, %				<0.001				<0.001
Married	2866 (60.3)	3198 (67.1)	3224 (70.7)		2578 (49.3)	2619 (54.7)	2758 (57.6)	
Others	1886 (39.7)	1568 (32.9)	1333 (29.3)		2651 (50.7)	2165 (45.3)	2032 (42.4)	
Educational attainments, %				<0.001				<0.001
Secondary high school or below	2829 (59.6)	2444 (51.3)	1758 (38.6)		2707 (51.8)	2283 (47.8)	1892 (39.5)	
College degree or above	1920 (40.4)	2322 (48.7)	2799 (61.4)		2519 (48.2)	2499 (52.2)	2893 (60.5)	
Smoking, %	2778 (58.5)	2666 (55.9)	2193 (48.1)	<0.001	2406 (46.0)	1675 (35.0)	1288 (26.9)	<0.001

Characteristic	The CDI scores							
	Male				Female			
	Tertile 1	Tertile 2	Tertile 3	*P*	Tertile 1	Tertile 2	Tertile 3	*P*
Drinking, %	4063 (85.5)	3817 (80.0)	3266 (71.7)	<0.001	3470 (66.3)	2548 (53.2)	2214 (46.2)	<0.001
Tea, %	1026 (21.6)	1108 (23.2)	1094 (24.0)	0.017	1324 (25.3)	1284 (26.8)	1359 (28.4)	0.005
Physical activity, weekly or daily MET-hour [d]	10.0 (6.7, 32.9)	10.0 (5.7, 28.6)	10.0 (5.7, 22.9)	<0.001	10.0 (5.0, 12.9)	10.0 (4.5, 12.2)	10.0 (4.7, 13.6)	0.109
BMI, kg/m^2	28.1 (24.6, 32.3)	28.0 (24.9, 31.6)	27.3 (24.4, 30.8)	<0.001	29.2 (24.5, 35.0)	28.7 (24.3, 33.8)	27.3 (23.4, 32.0)	<0.001
Waist Circumference, cm	100.7 (90.5, 111.4)	100.3 (91.1, 110.2)	98.7 (90.2, 108.2)	<0.001	97.5 (85.9, 110.0)	96.3 (85.6, 108.0)	93.3 (83.8, 103.9)	<0.001
Metabolic syndrome, %	1227 (31.5)	1219 (31.4)	1065 (28.0)	0.001	1541 (37.3)	1485 (39.1)	1288 (33.2)	<0.001
Abdominal obesity, %	2153 (46.8)	2103 (45.6)	1801 (41.1)	<0.001	3569 (71.0)	3219 (70.3)	2958 (64.6)	<0.001
Hyperglycemia, %	884 (36.5)	923 (38.6)	902 (38.5)	0.282	861 (34.5)	939 (38.5)	849 (35.3)	0.013
Hypertension, %	1578 (34.8)	1757 (38.3)	1725 (39.2)	<0.001	1766 (35.1)	1830 (39.6)	1819 (39.0)	<0.001
High TG, %	711 (31.8)	696 (32.4)	548 (26.7)	<0.001	535 (23.0)	547 (24.8)	479 (21.7)	0.070
Low HDL-C, %	1391 (30.8)	1305 (28.7)	1105 (25.4)	<0.001	1910 (38.8)	1601 (35.2)	1328 (29.0)	<0.001

Note:

[a] Data are presented as n (%) for categorical variables and as mean (standard deviation, SD) or median (interquartile range, IQR) for continuous variables, as appropriate. "-" indicates data not available.

[b] GNHS: Low income, < 3000.0 yuan / month. High income, >= 3000.0 yuan/month.

TCLSIH: Low income, < 10, 000 yuan / month. High income, >= 10, 000 yuan/month.

[c] PIR, the ratio of family income to poverty.

[d] Weekly MET-hours in the TCLSIH cohort, daily MET-hours in the GNHS cohort and the NHANES.

Abbreviations: BMI, body mass index; GNHS, Guangzhou Nutrition and Health Study; MET, metabolic equivalent of task; HDL-C, high-density lipoprotein cholesterol; NHANES, the Na-tional Health and Nutrition Examination Survey; PIR, the ratio of family income to poverty; TCLSIH, Tianjin Chronic Low-grade Systemic Inflammation and Health; TG, triglycerides.

Table S3. The CDI scores of the study participants across three studies [a]

Contents of CDI	GNHS N = 4025			TCLSIH N = 29165			NHANES N = 28890		
	Score	Male	Female	Score	Male	Female	Score	Male	Female
**CDI score**	100	57.0 (51.0, 63.5)	59.0 (53.0, 65.0)	93	51.0 (44.5, 58.0)	54.0 (47.0, 60.5)	98	48.0 (41.0, 56.5)	50.0 (42.0, 58.5)
C1. Wide varieties and balanced ingredients of foods	15	9.0 (7.0, 13.0)	11.0 (9.0, 13.0)	15	11.0 (7.0, 13.0)	11.0 (7.0, 13.0)	15	10.0 (7.0, 13.0)	10.0 (7.0, 13.0)
C2. Sufficient vegetables and plentiful fruits	20	12.0 (9.0, 14.0)	14.0 (12.0, 17.0)	20	11.0 (8.0, 14.0)	12.0 (10.0, 15.0)	20	10.0 (7.0, 12.0)	10.0 (8.0, 13.0)
C3. Ample fish and shellfish, moderate meat, poultry, eggs, and dairy products	15	7.0 (5.0, 9.0)	8.0 (6.0, 10.0)	15	8.0 (6.0, 10.0)	9.0 (7.0, 10.0)	15	8.0 (6.0, 10.0)	8.0 (7.0, 10.0)
C4. Regular consumption of beans, whole grain, nuts, and seeds	15	7.5 (4.0, 10.0)	8.0 (6.0, 11.5)	15	8.0 (4.0, 11.5)	8.0 (6.0, 11.5)	15	4.0 (0.0, 7.5)	6.0 (0.0, 8.0)
C5. Fresh ingredients and light cooking style, low sodium, and oil	20	12.0 (9.0, 15.0)	9.0 (7.0, 12.0)	20	11.0 (7.0, 14.0)	11.0 (7.0, 14.0)	20	11.0 (8.0, 14.0)	11.0 (8.0, 13.0) *
C6. More tea, less alcohol	6	4.0 (4.0, 6.0)	4.0 (4.0, 4.0)	6	2.0 (2.0, 2.0)	3.0 (2.0, 4.0)	6	0.0 (0.0, 0.0)	0.0 (0.0, 4.0)
C7. Cooking more by steaming, boiling, stewing, and quick stir-frying, less frying, preserving or pickling	5	1.0 (1.0, 5.0)	1.0 (1.0, 1.0)	0	0.0 (0.0, 0.0)	0.0 (0.0, 0.0)	5	1.0 (1.0, 5.0)	1.0 (1.0, 5.0)
C8. Enjoyment of "dimsum" and tea in the morning, frequent consumption of Cantonese-style soup, and paying attention to dietary regimen	4	2.0 (1.0, 2.0)	2.0 (2.0, 4.0)	2	0.0 (0.0, 0.0)	0.0 (0.0, 2.0)	2	0.0 (0.0, 2.0)	2.0 (0.0, 2.0)
**aMED score**	8	4.0 (2.5, 5.0)	4.0 (2.5, 5.0)	8	4.0 (3.0, 6.0)	4.0 (3.0, 5.0)	8	5.5 (5.0, 6.5)	6.0 (5.0, 6.5)
**DASH score**	40	23.3 (20.5, 26.0)	23.4 (21.5, 26.0)	40	24.0 (21.0, 27.0)	24.0 (22.0, 27.0)	40	26.5 (24.5, 28.5)	27.0 (25.0, 29.0)
**DBI-LBS**	72	15.0 (12.0, 19.0)	12.5 (9.5, 16.0)	72	14.0 (11.0, 18.0)	14.0 (10.0, 17.0)	72	42.0 (38.0, 46.0)	40.0 (35.0, 44.0)
**DBI-DQD**	96	39.5 (36.5, 42.0)	37.5 (35.5, 40.0)	96	32.0 (26.0, 37.0)	29.0 (24.0, 34.0)	96	54.0 (48.0, 58.0)	50.0 (44.0, 54.0)

[a] Data are presented as medians (interquartile ranges) for continuous variables.

* Non-significant.

Abbreviations: aMED, alternate Mediterranean Diet score; CDI, Cantonese dietary index; C1-C8, facets of CDI; DASH, Dietary Approaches to Stop Hypertension; DBI, Diet Balance Index; DBI-DQD, the dietary quality difference from DBI; DBI-LBS, low bound score from DBI; GNHS, Guangzhou Nutrition and Health Study; NHANES, the National Health and Nutrition Examination Survey; TCLSIH, Tianjin Chronic Low-grade Systemic Inflammation and Health.

Table S4. Components scores by tertiles between different sex in the GNHS, the TCLSIH, and the NHANES study [a]

Characteristic	Range	The CDI scores					
		Male			Female		
		Tertile 1	Tertile 2	Tertile 3	Tertile 1	Tertile 2	Tertile 3
GNHS							
Number of Participants		448	415	417	959	891	895
Range (min, max)		30.0, 53.0	53.1, 61.0	61.1, 84.5	27.0, 55.0	55.1, 63.0	63.1, 84.5
Total CDI	7 ~ 100	48.0 (45.0, 51.0)	57.0 (55.0, 59.0)	66.0 (63.5, 70.0)	50.0 (46.5, 53.0)	59.0 (57.5, 61.0)	68.0 (65.0, 71.5)
C1:	0 ~ 15	7.0 (7.0, 9.0)	9.0 (7.0, 13.0)	13.0 (11.0, 15.0)	9.0 (7.0, 9.0)	11.0 (9.0, 13.0)	13.0 (11.0, 15.0)
C11: dietary variety	0 ~ 10	2.0 (2.0, 4.0)	4.0 (2.0, 8.0)	8.0 (6.0, 10.0)	4.0 (2.0, 4.0)	6.0 (4.0, 8.0)	8.0 (8.0, 10.0)
C12: breakfast frequency	0 ~ 5	5.0 (5.0, 5.0)	5.0 (5.0, 5.0)	5.0 (5.0, 5.0)	5.0 (5.0, 5.0)	5.0 (5.0, 5.0)	5.0 (5.0, 5.0)
C2	3 ~ 20	10.0 (8.0, 12.0)	11.0 (10.0, 14.0)	13.0 (11.0, 16.0)	12.0 (10.0, 15.0)	15.0 (12.0, 17.0)	16.0 (14.0, 18.0)
C21: total vegetables	1 ~ 5	2.0 (1.0, 3.0)	2.0 (1.0, 4.0)	3.0 (2.0, 4.0)	3.0 (2.0, 4.0)	3.0 (2.0, 4.0)	4.0 (3.0, 5.0)
C22: dark veg / total veg	0, 5	5.0 (5.0, 5.0)	5.0 (5.0, 5.0)	5.0 (5.0, 5.0)	5.0 (5.0, 5.0)	5.0 (5.0, 5.0)	5.0 (5.0, 5.0)
C23: fruits	2 ~ 10	4.0 (2.0, 4.0)	4.0 (2.0, 6.0)	6.0 (4.0, 8.0)	4.0 (4.0, 8.0)	8.0 (4.0, 8.0)	8.0 (6.0, 10.0)
C3	3 ~ 15	6.0 (4.0, 7.0)	7.0 (6.0, 9.0)	9.0 (7.0, 10.0)	7.0 (5.0, 8.0)	8.0 (6.0, 10.0)	10.0 (8.0, 11.0)
C31: total animal foods	1, 3, 5	1.0 (1.0, 3.0)	3.0 (1.0, 3.0)	3.0 (1.0, 3.0)	3.0 (1.0, 3.0)	3.0 (1.0, 3.0)	3.0 (1.0, 3.0)
C32: fish and poultry / animal	1 ~ 5	2.0 (1.0, 4.0)	3.0 (1.0, 4.0)	4.0 (2.0, 5.0)	2.0 (1.0, 4.0)	3.0 (2.0, 4.0)	4.0 (3.0, 5.0)
C33: dairy	1 ~ 5	1.0 (1.0, 2.0)	1.0 (1.0, 2.0)	2.0 (1.0, 3.0)	2.0 (1.0, 3.0)	2.0 (1.0, 3.0)	3.0 (2.0, 5.0)
C4	0 ~ 15	4.0 (2.0, 6.0)	7.5 (6.0, 10.0)	10.0 (8.0, 12.0)	4.0 (2.0, 8.0)	8.0 (6.0, 11.5)	11.5 (9.5, 13.5)
C41: whole grains and beans	0 ~ 7.5	0.0 (0.0, 4.0)	4.0 (2.0, 6.0)	6.0 (4.0, 7.5)	2.0 (0.0, 4.0)	4.0 (2.0, 6.0)	6.0 (4.0, 7.5)
C42: soybeans and nuts	0 ~ 7.5	2.0 (0.0, 4.0)	4.0 (2.0, 6.0)	6.0 (4.0, 6.0)	2.0 (0.0, 4.0)	4.0 (2.0, 6.0)	6.0 (4.0, 7.5)
C5	0 ~ 20	12.0 (8.0, 14.0)	12.0 (9.0, 16.0)	13.0 (11.0, 16.0)	9.0 (6.0, 12.0)	9.0 (7.0, 12.0)	11.0 (8.0, 13.0)
C51: SFA	0 ~ 7	4.0 (0.0, 6.0)	4.0 (2.0, 7.0)	6.0 (4.0, 7.0)	2.0 (0.0, 6.0)	4.0 (2.0, 6.0)	6.0 (2.0, 7.0)
C52: salt	0 ~ 7	4.0 (2.0, 6.0)	4.0 (2.0, 6.0)	6.0 (4.0, 6.0)	2.0 (0.0, 4.0)	4.0 (2.0, 6.0)	4.0 (2.0, 6.0)

Characteristic	Range	The CDI scores					
		Male			Female		
		Tertile 1	Tertile 2	Tertile 3	Tertile 1	Tertile 2	Tertile 3
C53: added sugar	0 ~ 6	5.0 (3.0, 6.0)	5.0 (3.0, 6.0)	3.0 (1.0, 5.0)	3.0 (1.0, 5.0)	1.0 (0.0, 5.0)	1.0 (0.0, 3.0)
**C6**	0 ~ 6	4.0 (4.0, 4.0)	4.0 (4.0, 6.0)	4.0 (4.0, 6.0)	4.0 (4.0, 4.0)	4.0 (4.0, 4.0)	4.0 (4.0, 4.0)
C61: tea	0, 2	0.0 (0.0, 2.0)	0.0 (0.0, 2.0)	0.0 (0.0, 2.0)	0.0 (0.0, 0.0)	0.0 (0.0, 0.0)	0.0 (0.0, 0.0)
C62: alcohol	0 ~ 4	4.0 (4.0, 4.0)	4.0 (4.0, 4.0)	4.0 (4.0, 4.0)	4.0 (4.0, 4.0)	4.0 (4.0, 4.0)	4.0 (4.0, 4.0) *
**C7**	1 ~ 5	1.0 (1.0, 3.0)	1.0 (1.0, 5.0)	1.0 (1.0, 5.0)	1.0 (1.0, 1.0)	1.0 (1.0, 1.0)	1.0 (1.0, 1.0)
**C8**	0 ~ 4	2.0 (0.0, 2.0)	2.0 (2.0, 2.0)	2.0 (2.0, 4.0)	2.0 (2.0, 2.0)	2.0 (2.0, 4.0)	2.0 (2.0, 4.0)
C81: food and medicine	0, 2	0.0 (0.0, 2.0)	0.0 (0.0, 2.0)	2.0 (0.0, 2.0)	0.0 (0.0, 2.0)	2.0 (0.0, 2.0)	2.0 (0.0, 2.0)
C82: dietary supple	0, 2	0.0 (0.0, 2.0)	2.0 (0.0, 2.0)	2.0 (0.0, 2.0)	2.0 (0.0, 2.0)	2.0 (0.0, 2.0)	2.0 (2.0, 2.0)
**TCLSIH**							
Number of Participants		5665	5101	5381	4392	4444	4182
Range (min, max)		15.0, 47.0	47.5, 55.5	26.0, 85.0	15.0, 49.0	49.5, 58.0	58.5, 83.0
**Total CDI**	6 ~ 93	42.0 (37.0, 45.0)	51.5.0 (49.5, 53.5)	61.0 (58.0, 64.5)	44.0 (39.0, 47.0)	54.0 (52.0, 56.0)	63.5 (60.5, 67.0)
**C1**	0 ~ 10	7.0 (7.0, 11.0)	11.0 (9.0, 13.0)	13.0 (11.0, 15.0)	7.0 (7.0, 9.0)	11.0 (9.0, 13.0)	13.0 (11.0, 15.0)
C11: dietary variety	0 ~ 10	4.0 (2.0, 6.0)	6.0 (4.0, 8.0)	8.0 (6.0, 10.0)	2.0 (2.0, 4.0)	6.0 (4.0, 8.0)	8.0 (6.0, 10.0)
C12: breakfast frequency	0 ~ 5	5.0 (5.0, 5.0)	5.0 (5.0, 5.0)	5.0 (5.0, 5.0)	5.0 (5.0, 5.0)	5.0 (5.0, 5.0)	5.0 (5.0, 5.0)
**C2**	3 ~ 20	9.0 (6.0, 12.0)	11.0 (9.0, 14.0)	13.0 (11.0, 16.0)	10.0 (7.0, 13.0)	12.0 (10.0, 15.0)	15.0 (12.0, 17.0)
C21: total vegetables	1 ~ 5	2.0 (1.0, 4.0)	3.0 (2.0, 4.0)	4.0 (3.0, 5.0)	2.0 (1.0, 4.0)	3.0 (2.0, 4.0)	4.0 (3.0, 5.0)
C22: dark veg / total veg	0, 5	0.0 (0.0, 5.0)	5.0 (0.0, 5.0)	5.0 (0.0, 5.0)	0.0 (0.0, 5.0)	5.0 (0.0, 5.0)	5.0 (0.0, 5.0)
C23: fruits	2 ~ 10	4.0 (2.0, 6.0)	6.0 (4.0, 8.0)	6.0 (4.0, 8.0)	6.0 (4.0, 8.0)	6.0 (4.0, 10.0)	8.0 (6.0, 10.0)
**C3**	3 ~ 15	7.0 (5.0, 9.0)	8.0 (7.0, 10.0)	9.0 (8.0, 11.0)	8.0 (6.0, 9.0)	9.0 (7.0, 10.0)	10.0 (8.0, 11.0)
C31: total animal foods	1, 3, 5	1.0 (1.0, 3.0)	3.0 (1.0, 3.0)	3.0 (1.0, 3.0)	3.0 (1.0, 3.0)	3.0 (1.0, 3.0)	3.0 (1.0, 3.0)

Characteristic	Range	The CDI scores					
		Male			Female		
		Tertile 1	Tertile 2	Tertile 3	Tertile 1	Tertile 2	Tertile 3
C32: fish and poultry / animal	1 ~ 5	2.0 (1.0, 4.0)	3.0 (2.0, 4.0)	4.0 (3.0, 5.0)	2.0 (1.0, 4.0)	3.0 (2.0, 4.0)	4.0 (2.0, 5.0)
C33: dairy	1 ~ 5	2.0 (1.0, 4.0)	3.0 (2.0, 4.0)	3.0 (2.0, 4.0)	3.0 (2.0, 4.0)	3.0 (2.0, 4.0)	4.0 (3.0, 5.0)
**C4**	0 ~ 15	4.0 (2.0, 7.5)	8.0 (6.0, 10.0)	11.5.0 (8.0, 13.5)	4.0 (2.0, 7.5)	8.0 (6.0, 11.5)	11.5 (9.5, 13.5)
C41: whole grains and beans	0 ~ 7.5	2.0 (0.0, 4.0)	4.0 (2.0, 6.0)	6.0 (4.0, 7.5)	2.0 (0.0, 4.0)	4.0 (2.0, 6.0)	6.0 (4.0, 7.5)
C42: soybeans and nuts	0 ~ 7.5	2.0 (0.0, 4.0)	4.0 (2.0, 6.0)	6.0 (4.0, 7.5)	2.0 (0.0, 4.0)	4.0 (2.0, 6.0)	6.0 (4.0, 7.5)
**C5**	0 ~ 20	8.0 (5.0, 12.0)	11.0 (7.0, 14.0)	13.0 (10.0, 16.0)	7.0 (4.0, 11.0)	11.0 (7.0, 14.0)	13.0 (10.0, 16.0)
C51: SFA	0 ~ 7	2.0 (0.0, 6.0)	4.0 (2.0, 6.0)	4.0 (2.0, 6.0)	4.0 (0.0, 6.0)	4.0 (2.0, 6.0)	6.0 (2.0, 6.0)
C52: salt	0 ~ 7	2.0 (0.0, 4.0)	4.0 (2.0, 6.0)	6.0 (4.0, 7.0)	2.0 (0.0, 4.0)	4.0 (2.0, 6.0)	6.0 (4.0, 7.0)
C53: added sugar	0 ~ 6	3.0 (1.0, 5.0)	3.0 (1.0, 5.0)	5.0 (1.0, 5.0)	1.0 (0.0, 3.0)	3.0 (1.0, 5.0)	3.0 (1.0, 5.0)
**C6**	0 ~ 6	2.0 (2.0, 2.0)	2.0 (2.0, 2.0)	2.0 (2.0, 2.0)	3.0 (2.0, 4.0)	3.0 (2.0, 4.0)	4.0 (2.0, 4.0)
C61: tea	0, 2	0.0 (0.0, 0.0)	0.0 (0.0, 0.0)	0.0 (0.0, 0.0)	0.0 (0.0, 0.0)	0.0 (0.0, 0.0)	0.0 (0.0, 0.0)
C62: alcohol	0 ~ 4	2.0 (2.0, 2.0)	2.0 (2.0, 2.0)	2.0 (2.0, 2.0)	3.0 (2.0, 4.0)	3.0 (2.0, 4.0)	4.0 (2.0, 4.0)
**C7**	0	0.0 (0.0, 0.0)	0.0 (0.0, 0.0)	0.0 (0.0, 0.0)	0.0 (0.0, 0.0)	0.0 (0.0, 0.0)	0.0 (0.0, 0.0)
**C8**	0 ~ 2	0.0 (0.0, 0.0)	0.0 (0.0, 0.0)	0.0 (0.0, 2.0)	0.0 (0.0, 0.0)	0.0 (0.0, 2.0)	0.0 (0.0, 2.0)
C81: food and medicine	0	0.0 (0.0, 0.0)	0.0 (0.0, 0.0)	0.0 (0.0, 0.0)	0.0 (0.0, 0.0)	0.0 (0.0, 0.0)	0.0 (0.0, 0.0)
C82: dietary supple	0, 2	0.0 (0.0, 0.0)	0.0 (0.0, 0.0)	0.0 (0.0, 2.0)	0.0 (0.0, 0.0)	0.0 (0.0, 2.0)	0.0 (0.0, 2.0)
**NHANES**							
Number of Participants		4754	4769	4558	5230	4787	4792
Range (min, max)		14.0, 43.0	43.1, 53.5	53.6, 89.0	16.0, 45.0	45.1, 55.5	55.6, 89.0
**Total CDI**	7 ~ 98	38.0 (34.0, 41.0)	48.0 (46.0, 51.0)	60.5 (57.0, 65.5)	39.0 (35.0, 42.0)	50.5 (48.0, 53.0)	62.5 (59.0, 68.0)
**C1**	0 ~ 15	7.0 (7.0, 10.0)	10.0 (7.0, 13.0)	13.0 (10.0, 15.0)	7.0 (7.0, 10.0)	10.0 (7.0, 13.0)	13.0 (10.0, 15.0)
C11: dietary variety	0 ~ 10	2.0 (2.0, 5.0)	8.0 (5.0, 10.0)	8.0 (5.0, 10.0)	2.0 (2.0, 5.0)	5.0 (2.0, 8.0)	8.0 (5.0, 10.0)

Characteristic	Range	The CDI scores					
		Male			Female		
		Tertile 1	Tertile 2	Tertile 3	Tertile 1	Tertile 2	Tertile 3
C12: breakfast frequency	0 ~ 5	5.0 (5.0, 5.0)	5.0 (5.0, 5.0)	5.0 (5.0, 5.0)	5.0 (5.0, 5.0)	5.0 (5.0, 5.0)	5.0 (5.0, 5.0)
**C2**	3 ~ 20	8.0 (5.0, 10.0)	10.0 (8.0, 12.0)	13.0 (10.0, 15.0)	8.0 (6.0, 10.0)	10.0 (8.0, 13.0)	14.0 (11.0, 16.0)
C21: total vegetables	1 ~ 5	2.0 (1.0, 4.0)	3.0 (2.0, 4.0)	4.0 (3.0, 5.0)	2.0 (1.0, 3.0)	3.0 (2.0, 4.0)	4.0 (2.0, 5.0)
C22: dark veg/total veg	0, 5	0.0 (0.0, 5.0)	5.0 (0.0, 5.0)	5.0 (0.0, 5.0)	0.0 (0.0, 5.0)	5.0 (0.0, 5.0)	5.0 (0.0, 5.0)
C23: fruits	2 ~ 10	2.0 (2.0, 4.0)	4.0 (2.0, 6.0)	6.0 (4.0, 8.0)	2.0 (2.0, 4.0)	4.0 (2.0, 6.0)	6.0 (4.0, 8.0)
**C3**	3 ~ 15	7.0 (5.0, 9.0)	8.0 (7.0, 10.0)	9.0 (7.0, 11.0)	7.0 (6.0, 9.0)	9.0 (7.0, 10.0)	10.0 (8.0, 11.0)
C31: total animal foods	1, 3, 5	3.0 (1.0, 3.0)	3.0 (1.0, 3.0)	3.0 (1.0, 5.0)	3.0 (1.0, 3.0)	3.0 (1.0, 3.0)	3.0 (1.0, 5.0)
C32: fish and poultry / animal	1 ~ 5	1.0 (1.0, 4.0)	2.0 (1.0, 4.0)	3.0 (1.0, 4.0)	2.0 (1.0, 4.0)	3.0 (1.0, 4.0)	4.0 (1.0, 5.0)
C33: dairy	1 ~ 5	2.0 (1.0, 3.0)	3.0 (2.0, 4.0)	3.0 (2.0, 5.0)	3.0 (1.0, 4.0)	3.0 (2.0, 4.0)	4.0 (3.0, 5.0)
**C4**	0 ~ 15	0.0 (0.0, 2.0)	4.0 (0.0, 7.5)	8.0 (6.0, 13.5)	0.0 (0.0, 4.0)	6.0 (2.0, 7.5)	9.5 (7.5, 13.5)
C41: whole grains and beans	0 ~ 7.5	0.0 (0.0, 0.0)	0.0 (0.0, 4.0)	6.0 (2.0, 7.5)	0.0 (0.0, 2.0)	2.0 (0.0, 6.0)	6.0 (2.0, 7.5)
C42: soybeans and nuts	0 ~ 7.5	0.0 (0.0, 0.0)	0.0 (0.0, 6.0)	6.0 (0.0, 7.5)	0.0 (0.0, 0.0)	0.0 (0.0, 6.0)	6.0 (0.0, 7.5)
**C5**	0 ~ 20	9.0 (6.0, 11.0)	11.0 (8.0, 13.0)	13.0 (10.0, 15.0)	9.0 (6.0, 11.0)	11.0 (8.0, 13.0)	13.0 (11.0, 15.0)
C51: SFA	0 ~ 7	2.0 (0.0, 6.0)	4.0 (2.0, 6.0)	6.0 (4.0, 7.0)	2.0 (0.0, 6.0)	4.0 (2.0, 6.0)	6.0 (4.0, 7.0)
C52: salt	0 ~ 7	4.0 (0.0, 6.0)	4.0 (2.0, 6.0)	4.0 (2.0, 6.0)	4.0 (0.0, 6.0)	4.0 (2.0, 6.0)	4.0 (2.0, 6.0)
C53: added sugar	0 ~ 6	1.0 (0.0, 5.0)	3.0 (1.0, 5.0)	5.0 (3.0, 6.0)	1.0 (0.0, 5.0)	3.0 (1.0, 5.0)	5.0 (3.0, 6.0)
**C6**	0 ~ 6	0.0 (0.0, 0.0)	0.0 (0.0, 0.0)	0.0 (0.0, 4.0)	0.0 (0.0, 4.0)	0.0 (0.0, 4.0)	4.0 (0.0, 4.0)
C61: tea	0, 2	0.0 (0.0, 0.0)	0.0 (0.0, 0.0)	0.0 (0.0, 0.0)	0.0 (0.0, 0.0)	0.0 (0.0, 0.0)	0.0 (0.0, 0.0)
C62: alcohol	0 ~ 4	0.0 (0.0, 0.0)	0.0 (0.0, 0.0)	0.0 (0.0, 4.0)	0.0 (0.0, 4.0)	0.0 (0.0, 4.0)	4.0 (0.0, 4.0)
**C7**	1 ~ 5	1.0 (1.0, 5.0)	1.0 (1.0, 5.0)	3.0 (1.0, 5.0)	1.0 (1.0, 5.0)	1.0 (1.0, 5.0)	3.0 (1.0, 5.0)
**C8**	0 ~ 2	0.0 (0.0, 2.0)	0.0 (0.0, 2.0)	2.0 (0.0, 2.0)	0.0 (0.0, 2.0)	2.0 (0.0, 2.0)	2.0 (0.0, 2.0)
C81: food and medicine	0	0.0 (0.0, 0.0)	0.0 (0.0, 0.0)	0.0 (0.0, 0.0)	0.0 (0.0, 0.0)	0.0 (0.0, 0.0)	0.0 (0.0, 0.0)

Characteristic	Range	The CDI scores					
		Male			Female		
		Tertile 1	Tertile 2	Tertile 3	Tertile 1	Tertile 2	Tertile 3
C82: dietary supplements	0, 2	0.0 (0.0, 2.0)	0.0 (0.0, 2.0)	2.0 (0.0, 2.0)	0.0 (0.0, 2.0)	2.0 (0.0, 2.0)	2.0 (0.0, 2.0)

Note: * non-significant.

[a] Date are presented as median (IQR), unless noted.

Abbreviations: CDI, Cantonese dietary index; C1-C8, facet 1-8 of the CDI. GNHS, Guangzhou Nutrition and Health Study. NHANES, the National Health and Nutrition Examination Survey. SFA, saturated fat. TCLSIH, Tianjin Chronic Low-grade Systemic Inflammation and Health.

Table S5. The intra-class correlation coefficients of the CDI score between the baseline and 3rd follow up in the GNHS cohort (n = 2525) [a]

Variables	ICC	*P*
Total CDI	0.332	< 0.001
C1	0.029	0.074
C2	0.273	< 0.001
C3	0.234	< 0.001
C4	0.307	< 0.001
C5	0.261	< 0.001
C6	0.424	< 0.001
C7	0.999	< 0.001
C8	0.622	< 0.001

Note:

[a] Data are presented as intra-class correlation coefficients and its P values.

Abbreviations: CDI, Cantonese dietary index; C1-C8, facets 1-8 of the CDI. GNHS, Guangzhou Nutrition and Health Study; ICC, intra-class correlation coefficients.

Table S6. Association between CDI and multiple disease in the GNHS cohort, the TCLSIH cohort and NHANES study [a]

Outcomes	Models	Cases/N	Continuous variable		Tertiles of CDI				
			Per 10 scores		T_2 *vs.* T_1		T_3 *vs.* T_1		*P* for trend [b]
			OR	*P*	OR	*P*	OR	*P*	
GNHS cohort									
**Metabolic syndrome**	M1	1148/3844	**0.79 (0.73, 0.85)**	**4.32×10^{-9}**	**0.80 (0.67, 0.94)**	**7.57×10^{-3}**	**0.65 (0.55, 0.77)**	**7.47×10^{-7}**	**6.81×10^{-7}**
	M2		**0.78 (0.72, 0.85)**	**9.13×10^{-9}**	**0.77 (0.65, 0.92)**	**4.04×10^{-3}**	**0.64 (0.53, 0.76)**	**1.10×10^{-6}**	**9.55×10^{-7}**
Abdominal Obesity	M1	1448/4016	**0.91 (0.84, 0.98)**	**8.62×10^{-3}**	1.00 (0.85, 1.17)	9.57×10^{-1}	**0.81 (0.69, 0.95)**	**1.03×10^{-2}**	**1.22×10^{-2}**
	M2		**0.90 (0.84, 0.97)**	**8.35×10^{-3}**	0.97 (0.82, 1.14)	6.75×10^{-1}	**0.80 (0.68, 0.95)**	**8.74×10^{-3}**	**9.67×10^{-3}**
Hyperglycemia	M1	339/3930	**0.86 (0.76, 0.98)**	**2.10×10^{-2}**	1.01 (0.77, 1.33)	9.32×10^{-1}	0.78 (0.58, 1.03)	8.23×10^{-2}	9.44×10^{-2}
	M2		**0.84 (0.72, 0.98)**	**2.31×10^{-2}**	1.00 (0.73, 1.37)	9.83×10^{-1}	0.72 (0.51, 1.01)	5.81×10^{-2}	6.83×10^{-2}
HBP	M1	1529/4015	0.96 (0.89, 1.04)	3.58×10^{-1}	1.06 (0.89, 1.25)	5.13×10^{-1}	0.93 (0.79, 1.11)	4.27×10^{-1}	4.54×10^{-1}
	M2		0.95 (0.87, 1.05)	3.17×10^{-1}	1.00 (0.82, 1.21)	9.87×10^{-1}	0.89 (0.73, 1.09)	2.72×10^{-1}	2.83×10^{-1}
Low HDL-C	M1	1199/3925	**0.90 (0.83, 0.97)**	**7.25×10^{-3}**	0.85 (0.72, 1.01)	6.08×10^{-2}	0.85 (0.72, 1.01)	6.43×10^{-2}	5.77×10^{-2}
	M2		**0.90 (0.83, 0.98)**	**1.40×10^{-2}**	0.86 (0.72, 1.02)	7.51×10^{-2}	0.86 (0.73, 1.03)	9.33×10^{-2}	8.59×10^{-2}
High TG	M1	1117/3925	**0.89 (0.82, 0.96)**	**4.03×10^{-3}**	0.88 (0.74, 1.04)	1.44×10^{-1}	0.85 (0.71, 1.00)	5.52×10^{-2}	5.20×10^{-2}
	M2		**0.89 (0.82, 0.97)**	**5.21×10^{-3}**	0.87 (0.73, 1.04)	1.24×10^{-1}	0.85 (0.71, 1.01)	6.59×10^{-2}	6.21×10^{-2}
TCLSIH cohort									
**Metabolic syndrome**	M1	7548 / 29165	**0.92 (0.90, 0.95)**	**2.57×10^{-8}**	**0.93 (0.87, 0.99)**	**2.88×10^{-2}**	**0.85 (0.80, 0.91)**	**6.26×10^{-6}**	**6.26×10^{-6}**
	M2		**0.95 (0.92, 0.98)**	**7.31×10^{-4}**	0.96 (0.89, 1.03)	2.23×10^{-1}	**0.91 (0.85, 0.98)**	**1.31×10^{-2}**	**1.31×10^{-2}**
Abdominal Obesity	M1	10634 / 29165	**0.92 (0.90, 0.95)**	**2.67×10^{-9}**	**0.93 (0.88, 1.00)**	**3.48×10^{-2}**	**0.84 (0.79, 0.90)**	**1.48×10^{-7}**	**1.55×10^{-7}**
	M2		**0.93 (0.91, 0.96)**	**1.17×10^{-6}**	0.95 (0.89, 1.01)	9.31×10^{-2}	**0.86 (0.81, 0.93)**	**2.48×10^{-5}**	**2.51×10^{-5}**
Hyperglycemia	M1	2260 / 29165	1.03 (0.98, 1.08)	2.59×10^{-1}	0.98 (0.87, 1.10)	7.29×10^{-1}	1.08 (0.97, 1.22)	1.63×10^{-1}	1.37×10^{-1}
	M2		**1.11 (1.05, 1.16)**	**9.74×10^{-5}**	1.06 (0.94, 1.19)	3.60×10^{-1}	1.27 (1.13, 1.43)	**9.68×10^{-5}**	**6.74×10^{-5}**
HBP	M1	8255 / 29165	0.99 (0.96, 1.02)	4.14×10^{-1}	0.99 (0.92, 1.07)	8.73×10^{-1}	0.99 (0.92, 1.07)	8.66×10^{-1}	8.67×10^{-1}

Outcomes	Models	Cases/N	Continuous variable		Tertiles of CDI				
			Per 10 scores		T_2 *vs.* T_1		T_3 *vs.* T_1		*P* for trend [b]
			OR	*P*	OR	*P*	OR	*P*	
	M2		1.00 (0.96, 1.03)	8.47×10^{-1}	1.01 (0.93, 1.09)	8.26×10^{-1}	1.02 (0.94, 1.10)	6.68×10^{-1}	6.68×10^{-1}
Low HDL-C	M1	7805 / 29165	0.98 (0.95, 1.01)	1.67×10^{-1}	0.96 (0.90, 1.03)	2.74×10^{-1}	0.97 (0.91, 1.04)	4.25×10^{-1}	4.17×10^{-1}
	M2		1.00 (0.97, 1.03)	8.56×10^{-1}	0.99 (0.92, 1.05)	6.89×10^{-1}	1.02 (0.95, 1.10)	5.21×10^{-1}	5.29×10^{-1}
High TG	M1	7578 / 29165	**0.93 (0.90, 0.96)**	**4.66×10^{-7}**	0.97 (0.90, 1.04)	3.36×10^{-1}	0.87 (0.81, 0.93)	1.13×10^{-4}	1.18×10^{-4}
	M2		**0.96 (0.93, 0.99)**	**2.26×10^{-2}**	1.01 (0.94, 1.08)	8.91×10^{-1}	0.95 (0.88, 1.03)	2.02×10^{-1}	2.04×10^{-1}
MASLD	M1	9860 / 29165	**0.91 (0.89, 0.94)**	**1.39×10^{-11}**	**0.91 (0.86, 0.97)**	**5.26×10^{-3}**	**0.83 (0.77, 0.88)**	**4.87×10^{-9}**	**4.92×10^{-9}**
	M2		**0.93 (0.90, 0.96)**	**7.11×10^{-7}**	**0.93 (0.87, 0.99)**	**3.13×10^{-2}**	**0.87 (0.81, 0.93)**	**3.15×10^{-5}**	**3.15×10^{-5}**
NHANES									
Metabolic syndrome	M1	7825 / 23383	**0.82 (0.80, 0.84)**	**1.43×10^{-50}**	**0.87 (0.81, 0.94)**	**1.81×10^{-4}**	**0.61 (0.57, 0.66)**	**2.13×10^{-40}**	**1.67×10^{-40}**
	M2		**0.84 (0.82, 0.87)**	**9.13×10^{-29}**	**0.88 (0.81, 0.95)**	**1.66×10^{-3}**	**0.65 (0.59, 0.70)**	**5.81×10^{-24}**	**7.23×10^{-24}**
Abdominal Obesity	M1	15803 / 27778	**0.82 (0.81, 0.84)**	**3.27×10^{-63}**	**0.85 (0.80, 0.90)**	**2.03×10^{-7}**	**0.62 (0.58, 0.66)**	**1.61×10^{-49}**	**2.18×10^{-49}**
	M2		**0.82 (0.80, 0.84)**	**1.53×10^{-58}**	**0.83 (0.78, 0.88)**	**1.11×10^{-8}**	**0.62 (0.58, 0.66)**	**7.33×10^{-46}**	**8.18×10^{-46}**
Hyperglycemia	M1	5358 / 14500	**0.93 (0.90, 0.96)**	**3.37×10^{-5}**	0.97 (0.88, 1.06)	4.76×10^{-1}	**0.85 (0.77, 0.93)**	**8.00×10^{-4}**	**7.31×10^{-4}**
	M2		**0.93 (0.89, 0.97)**	**1.39×10^{-3}**	0.96 (0.85, 1.08)	5.06×10^{-1}	**0.83 (0.73, 0.94)**	**4.04×10^{-3}**	**4.11×10^{-3}**
HBP	M1	10475 / 27832	**0.91 (0.89, 0.93)**	**2.29×10^{-12}**	0.94 (0.88, 1.01)	1.19×10^{-1}	0.79 (0.73, 0.85)	4.95×10^{-10}	3.90×10^{-10}
	M2		**0.92 (0.88, 0.96)**	**8.17×10^{-5}**	1.00 (0.90, 1.12)	9.92×10^{-1}	0.81 (0.72, 0.91)	3.22×10^{-4}	3.10×10^{-4}
Low HDL-C	M1	8640 / 27467	**0.91 (0.89, 0.93)**	**1.15×10^{-14}**	**0.92 (0.86, 0.98)**	**1.08×10^{-2}**	**0.82 (0.76, 0.87)**	**3.14×10^{-9}**	**3.35×10^{-9}**
	M2		**0.94 (0.92, 0.96)**	**6.75×10^{-7}**	0.95 (0.89, 1.02)	1.63×10^{-1}	**0.89 (0.83, 0.95)**	**6.94×10^{-4}**	**7.19×10^{-4}**
High TG	M1	3516 / 13176	**0.92 (0.89, 0.96)**	**1.54×10^{-5}**	1.03 (0.94, 1.14)	4.92×10^{-1}	**0.86 (0.78, 0.95)**	**3.88×10^{-3}**	**4.48×10^{-3}**
	M2		**0.96 (0.92, 0.99)**	**1.90×10^{-2}**	1.07 (0.97, 1.18)	1.53×10^{-1}	0.94 (0.85, 1.05)	2.64×10^{-1}	2.82×10^{-1}

Note: Logistic regression models for unscaled CDI scores (independent variable) and metabolism outcomes (dependent variable). M1, model 1, adjusted for age, and sex. M2, model 2, adjusted for age, sex, BMI (not included when abdominal obesity or MetS was outcome), educational attainments, marital status, smoking status, physical activity, antihypertensive medications, lipid-lowering medications, antidiabetic medications, and total energy intake.

[a] Data are presented as ORs (95% CI) and *P* values.

[b] *P* for trend was calculated by using the median value of each tertile as a continuous exposure.

Abbreviations: CDI, Canton-ese dietary index; CI, confidence interval; GNHS, Guangzhou Nutrition and Health Study; HBP, hypertension; HDL-C, high-density lipoprotein cholesterol; MetS, metabolic syndrome; NHANES, the National Health and Nutrition Examination Survey; OR, odds ratio; TCLSIH, Tianjin Chronic Low-grade Systemic Inflammation and Health; TG, triglycerides.

Table S7. Association between diseases and diet-quality scores in the GNHS cohort, the TCLSIH cohort and NHANES study [a]

Outcomes	Models	CDI		aMED		DASH		DBI-LBS		DBI-DQD	
		OR (95% CI)	*P*	OR (95% CI)	*P*	OR (95% CI)	*P*	OR (95% CI)	*P*	OR (95%CI)	*P*
**GNHS cohort**											
**Metabolic syndrome**	M1	0.81 (0.75, 0.87)	4.32×10^{-9}	0.91 (0.85, 0.98)	9.15×10^{-3}	0.82 (0.77, 0.89)	1.37×10^{-7}	1.15 (1.07, 1.23)	1.42×10^{-4}	1.14 (1.07, 1.23)	2.46×10^{-4}
	M2	0.80 (0.74, 0.86)	9.13×10^{-9}	0.91 (0.84, 0.99)	2.85×10^{-2}	0.83 (0.77, 0.90)	2.90×10^{-6}	1.13 (1.05, 1.22)	1.70×10^{-3}	1.12 (1.04, 1.21)	4.24×10^{-3}
Abdominal Obesity	M1	0.92 (0.86, 0.98)	8.62×10^{-3}	1.04 (0.98, 1.11)	1.94×10^{-1}	0.88 (0.82, 0.94)	1.40×10^{-4}	1.06 (0.99, 1.13)	7.77×10^{-2}	1.08 (1.01, 1.15)	3.10×10^{-2}
	M2	0.91 (0.85, 0.98)	8.35×10^{-3}	1.07 (0.99, 1.15)	9.60×10^{-2}	0.89 (0.83, 0.95)	9.13×10^{-4}	1.04 (0.96, 1.11)	3.47×10^{-1}	1.05 (0.98, 1.13)	1.74×10^{-1}
Hyperglycemia	M1	0.87 (0.78, 0.98)	2.10×10^{-2}	0.84 (0.75, 0.94)	2.71×10^{-3}	0.94 (0.84, 1.06)	3.22×10^{-1}	1.04 (0.93, 1.17)	4.62×10^{-1}	1.05 (0.94, 1.18)	4.13×10^{-1}
	M2	0.85 (0.74, 0.98)	2.31×10^{-2}	0.83 (0.72, 0.97)	1.88×10^{-2}	0.95 (0.82, 1.09)	4.36×10^{-1}	1.05 (0.91, 1.21)	5.34×10^{-1}	1.04 (0.90, 1.19)	6.18×10^{-1}
HBP	M1	0.97 (0.90, 1.04)	3.58×10^{-1}	0.94 (0.87, 1.00)	6.76×10^{-2}	0.93 (0.86, 0.99)	3.23×10^{-2}	1.06 (0.98, 1.13)	1.30×10^{-1}	1.05 (0.98, 1.13)	1.87×10^{-1}
	M2	0.96 (0.88, 1.04)	3.17×10^{-1}	0.95 (0.86, 1.04)	2.31×10^{-1}	0.93 (0.85, 1.01)	7.32×10^{-2}	1.05 (0.96, 1.14)	2.91×10^{-1}	1.05 (0.96, 1.14)	3.00×10^{-1}
Low HDL-C	M1	0.91 (0.85, 0.97)	7.25×10^{-3}	0.92 (0.86, 0.99)	2.32×10^{-2}	0.89 (0.83, 0.95)	1.21×10^{-3}	1.13 (1.06, 1.22)	5.84×10^{-4}	1.10 (1.02, 1.18)	9.43×10^{-3}
	M2	0.91 (0.85, 0.98)	1.40×10^{-2}	0.91 (0.84, 0.98)	1.65×10^{-2}	0.89 (0.83, 0.96)	3.01×10^{-3}	1.13 (1.05, 1.21)	1.86×10^{-3}	1.08 (1.01, 1.16)	3.59×10^{-2}
High TG	M1	0.90 (0.84, 0.97)	4.03×10^{-3}	0.92 (0.86, 0.99)	2.89×10^{-2}	0.89 (0.83, 0.96)	2.04×10^{-3}	1.17 (1.09, 1.25)	1.97×10^{-5}	1.14 (1.06, 1.22)	5.78×10^{-4}
	M2	0.90 (0.84, 0.97)	5.21×10^{-3}	0.92 (0.85, 0.99)	3.23×10^{-2}	0.90 (0.83, 0.97)	5.22×10^{-3}	1.17 (1.08, 1.26)	5.15×10^{-5}	1.12 (1.04, 1.21)	2.50×10^{-3}
**TCLSIH cohort**											
**Metabolic syndrome**	M1	0.92 (0.90, 0.95)	2.57×10^{-8}	0.96 (0.94, 0.99)	9.10×10^{-3}	0.90 (0.87, 0.93)	7.68×10^{-13}	1.07 (1.04, 1.10)	9.73×10^{-7}	1.12 (1.09, 1.16)	2.54×10^{-16}
	M2	0.95 (0.92, 0.98)	7.31×10^{-4}	0.99 (0.96, 1.02)	4.31×10^{-1}	0.92 (0.89, 0.95)	3.91×10^{-8}	1.05 (1.02, 1.08)	1.14×10^{-3}	1.09 (1.06, 1.13)	8.26×10^{-10}
Abdominal Obesity	M1	0.92 (0.90, 0.95)	2.67×10^{-9}	1.00 (0.97, 1.02)	8.77×10^{-1}	0.92 (0.89, 0.94)	5.59×10^{-10}	1.04 (1.01, 1.06)	4.49×10^{-3}	1.09 (1.07, 1.12)	1.75×10^{-11}
	M2	0.93 (0.91, 0.96)	1.17×10^{-6}	1.00 (0.97, 1.03)	7.80×10^{-1}	0.92 (0.90, 0.95)	3.46×10^{-8}	1.03 (1.00, 1.06)	5.04×10^{-2}	1.07 (1.04, 1.10)	3.05×10^{-7}
Hyperglycemia	M1	1.03 (0.98, 1.08)	2.59×10^{-1}	0.98 (0.94, 1.03)	4.83×10^{-1}	0.98 (0.93, 1.03)	4.06×10^{-1}	1.03 (0.98, 1.08)	2.03×10^{-1}	1.04 (0.99, 1.09)	1.04×10^{-1}
	M2	1.11 (1.05, 1.16)	9.74×10^{-5}	1.07 (1.02, 1.13)	1.18×10^{-2}	1.04 (0.99, 1.10)	1.13×10^{-1}	0.98 (0.94, 1.03)	4.95×10^{-1}	0.99 (0.95, 1.04)	8.01×10^{-1}

Outcomes	Models	CDI		aMED		DASH		DBI-LBS		DBI-DQD	
		OR (95% CI)	*P*	OR (95% CI)	*P*	OR (95% CI)	*P*	OR (95% CI)	*P*	OR (95%CI)	*P*
HBP	M1	0.99 (0.96, 1.02)	4.14×10^{-1}	0.96 (0.94, 0.99)	2.19×10^{-2}	0.97 (0.94, 1.00)	9.01×10^{-2}	1.04 (1.01, 1.07)	1.20×10^{-2}	1.05 (1.02, 1.08)	1.87×10^{-3}
	M2	1.00 (0.96, 1.03)	8.47×10^{-1}	0.97 (0.94, 1.01)	1.56×10^{-1}	0.98 (0.95, 1.01)	2.03×10^{-1}	1.03 (1.00, 1.06)	8.82×10^{-2}	1.04 (1.01, 1.08)	6.12×10^{-3}
Low HDL-C	M1	0.98 (0.95, 1.01)	1.67×10^{-1}	0.99 (0.96, 1.01)	2.97×10^{-1}	0.97 (0.94, 0.99)	2.03×10^{-2}	1.06 (1.03, 1.09)	1.80×10^{-5}	1.05 (1.02, 1.08)	7.51×10^{-4}
	M2	1.00 (0.97, 1.03)	8.56×10^{-1}	1.00 (0.97, 1.04)	7.74×10^{-1}	0.99 (0.96, 1.02)	3.40×10^{-1}	1.05 (1.02, 1.08)	4.20×10^{-4}	1.03 (1.00, 1.06)	3.66×10^{-2}
High TG	M1	0.93 (0.90, 0.96)	4.66×10^{-7}	0.92 (0.90, 0.95)	8.61×10^{-8}	0.90 (0.87, 0.92)	6.22×10^{-13}	1.06 (1.03, 1.09)	1.70×10^{-5}	1.12 (1.09, 1.16)	1.28×10^{-15}
	M2	0.96 (0.93, 0.99)	2.26×10^{-2}	0.94 (0.91, 0.98)	8.27×10^{-4}	0.93 (0.90, 0.96)	2.95×10^{-6}	1.04 (1.01, 1.08)	4.14×10^{-3}	1.09 (1.06, 1.12)	7.51×10^{-9}
**NHANES**											
**Metabolic syndrome**	M1	0.81 (0.78, 0.83)	4.86×10^{-45}	0.84 (0.81, 0.86)	1.11×10^{-31}	0.72 (0.70, 0.74)	3.11×10^{-94}	1.00 (0.97, 1.03)	8.76×10^{-1}	0.95 (0.92, 0.98)	3.21×10^{-3}
	M2	0.80 (0.77, 0.84)	2.10×10^{-19}	0.80 (0.76, 0.84)	4.52×10^{-21}	0.72 (0.69, 0.76)	2.59×10^{-39}	1.09 (1.04, 1.15)	6.72×10^{-4}	1.03 (0.97, 1.09)	3.16×10^{-1}
Abdominal Obesity	M1	0.83 (0.81, 0.85)	8.75×10^{-45}	0.85 (0.83, 0.87)	2.25×10^{-36}	0.78 (0.76, 0.81)	4.59×10^{-71}	1.05 (1.02, 1.08)	1.16×10^{-3}	1.04 (1.01, 1.06)	1.47×10^{-2}
	M2	0.82 (0.79, 0.85)	1.02×10^{-25}	0.83 (0.80, 0.86)	3.99×10^{-25}	0.77 (0.74, 0.80)	3.34×10^{-39}	1.09 (1.05, 1.14)	4.78×10^{-5}	1.06 (1.01, 1.11)	1.11×10^{-2}
Hyperglycemia	M1	0.91 (0.87, 0.94)	2.99×10^{-6}	1.04 (1.00, 1.09)	4.91×10^{-2}	0.85 (0.81, 0.88)	4.92×10^{-15}	0.85 (0.82, 0.89)	5.00×10^{-14}	0.81 (0.77, 0.84)	4.91×10^{-22}
	M2	0.91 (0.85, 0.98)	1.27×10^{-2}	1.02 (0.95, 1.09)	6.06×10^{-1}	0.88 (0.82, 0.95)	5.06×10^{-4}	1.02 (0.95, 1.10)	5.86×10^{-1}	0.95 (0.88, 1.03)	2.27×10^{-1}
HBP	M1	0.92 (0.89, 0.94)	2.07×10^{-8}	1.00 (0.97, 1.03)	8.47×10^{-1}	0.87 (0.85, 0.90)	2.19×10^{-17}	1.01 (0.98, 1.04)	4.78×10^{-1}	1.03 (0.99, 1.06)	1.23×10^{-1}
	M2	0.92 (0.86, 0.98)	1.44×10^{-2}	1.00 (0.94, 1.07)	9.23×10^{-1}	0.90 (0.84, 0.96)	2.64×10^{-3}	1.06 (0.99, 1.15)	1.09×10^{-1}	1.06 (0.98, 1.15)	1.32×10^{-1}
Low HDL-C	M1	0.87 (0.85, 0.90)	1.69×10^{-21}	0.81 (0.78, 0.83)	2.80×10^{-50}	0.83 (0.81, 0.86)	1.71×10^{-36}	1.02 (0.99, 1.05)	1.46×10^{-1}	0.93 (0.90, 0.96)	1.17×10^{-6}
	M2	0.89 (0.86, 0.93)	3.73×10^{-8}	0.80 (0.77, 0.83)	4.87×10^{-28}	0.84 (0.81, 0.88)	1.27×10^{-16}	1.05 (1.00, 1.10)	3.48×10^{-2}	0.95 (0.90, 0.99)	2.34×10^{-2}
High TG	M1	0.88 (0.84, 0.92)	2.05×10^{-9}	0.94 (0.90, 0.98)	2.57×10^{-3}	0.89 (0.85, 0.93)	3.47×10^{-8}	1.07 (1.03, 1.12)	1.60×10^{-3}	1.04 (1.00, 1.09)	6.85×10^{-2}
	M2	0.92 (0.86, 0.97)	3.50×10^{-3}	0.94 (0.88, 0.99)	2.13×10^{-2}	0.92 (0.87, 0.98)	7.46×10^{-3}	1.10 (1.03, 1.17)	6.46×10^{-3}	1.03 (0.96, 1.10)	3.78×10^{-1}

Note: Logistic regression models for z-score standardized dietary quality indices (independent variable) and metabolism outcomes (dependent variable). M1, model 1, adjusted for age, and sex. M2, model 2, adjusted for age, sex, BMI (not included when abdominal obesity or MetS was outcome), educational attainments, marital status, smoking status, physical activity, antihypertensive medications, lipid-lowering medications, antidiabetic medications, total energy intake, and dietary supplement use (except for CDI).

[a] Data are presented as ORs (95% CI) and P values.

Abbreviations: aMed, alternative Mediterranean dietary index; CDI, Can-tonese dietary index; CI, confidence interval; DASH, dietary approaches to stop hypertension; DBI, dietary balance index; DBI-LBS, low bound score of DBI. DBI-DQD, the dietary quality difference of DBI; GNHS, Guangzhou Nutrition and Health Study; HBP, hypertension; HDL-C, high-density lipoprotein cholesterol; MetS, metabolic syndrome; NHANES, the National Health and Nutrition Examination Survey; OR, odds ratio; TCLSIH, Tianjin Chronic Low-grade Systemic Inflammation and Health; TG, triglycerides.

Table S8. Association between diseases and domain scores of CDI across three cohort [a]

Outcomes	Models	CDI		Domain 1		Domain 2	
		OR (95% CI)	*P*	OR (95% CI)	*P*	OR (95% CI)	*P*
GNHS cohort							
Metabolic syndrome	M1	**0.81 (0.75, 0.87)**	**4.32×10^{-9}**	**0.84 (0.78, 0.90)**	**1.78×10^{-6}**	**0.83 (0.77, 0.90)**	**7.56×10^{-7}**
	M2	**0.80 (0.74, 0.86)**	**9.13×10^{-9}**	**0.85 (0.79, 0.92)**	**2.11×10^{-5}**	**0.80 (0.75, 0.87)**	**2.10×10^{-8}**
Abdominal Obesity	M1	**0.92 (0.86, 0.98)**	**8.62×10^{-3}**	**0.89 (0.84, 0.95)**	**6.66×10^{-4}**	1.00 (0.94, 1.07)	9.41×10^{-1}
	M2	**0.91 (0.85, 0.98)**	**8.35×10^{-3}**	**0.89 (0.83, 0.96)**	**1.44×10^{-3}**	0.99 (0.92, 1.06)	7.16×10^{-1}
Hyperglycemia	M1	**0.87 (0.78, 0.98)**	**2.10×10^{-2}**	**0.83 (0.74, 0.94)**	**2.01×10^{-3}**	1.01 (0.90, 1.14)	8.07×10^{-1}
	M2	**0.85 (0.74, 0.98)**	**2.31×10^{-2}**	0.90 (0.78, 1.03)	1.24×10^{-1}	**0.84 (0.73, 0.97)**	**1.47×10^{-2}**
HBP	M1	0.97 (0.90, 1.04)	3.58×10^{-1}	0.98 (0.91, 1.05)	5.29×10^{-1}	0.97 (0.90, 1.04)	3.26×10^{-1}
	M2	0.96 (0.88, 1.04)	3.17×10^{-1}	0.99 (0.91, 1.07)	7.60×10^{-1}	0.93 (0.85, 1.01)	8.34×10^{-2}
Low HDL-C	M1	**0.91 (0.85, 0.97)**	**7.25×10^{-3}**	1.00 (0.93, 1.07)	9.66×10^{-1}	**0.79 (0.74, 0.85)**	**4.88×10^{-10}**
	M2	**0.91 (0.85, 0.98)**	**1.40×10^{-2}**	1.01 (0.93, 1.08)	8.92×10^{-1}	**0.80 (0.74, 0.86)**	**4.82×10^{-9}**
High TG	M1	**0.90 (0.84, 0.97)**	**4.03×10^{-3}**	0.95 (0.88, 1.02)	1.40×10^{-1}	**0.87 (0.81, 0.93)**	**8.08×10^{-5}**
	M2	**0.90 (0.84, 0.97)**	**5.21×10^{-3}**	0.95 (0.88, 1.02)	1.65×10^{-1}	**0.86 (0.80, 0.93)**	**9.33×10^{-5}**
TCLSIH cohort							
Metabolic syndrome	M1	**0.92 (0.90, 0.95)**	**2.57×10^{-8}**	**0.94 (0.91, 0.97)**	**2.03×10^{-5}**	**0.92 (0.89, 0.94)**	**1.33×10^{-9}**
	M2	**0.95 (0.92, 0.98)**	**7.31×10^{-4}**	**0.96 (0.93, 0.99)**	**1.01×10^{-2}**	**0.94 (0.91, 0.97)**	**2.78×10^{-4}**
Abdominal Obesity	M1	**0.92 (0.90, 0.95)**	**2.67×10^{-9}**	**0.93 (0.90, 0.95)**	**3.44×10^{-8}**	**0.95 (0.92, 0.97)**	**5.41×10^{-5}**
	M2	**0.93 (0.91, 0.96)**	**1.17×10^{-6}**	**0.94 (0.91, 0.96)**	**4.03×10^{-6}**	**0.96 (0.93, 0.99)**	**6.13×10^{-3}**
Hyperglycemia	M1	1.03 (0.98, 1.08)	2.59×10^{-1}	1.04 (0.99, 1.09)	1.21×10^{-1}	0.99 (0.94, 1.03)	5.83×10^{-1}
	M2	**1.11 (1.05, 1.16)**	**9.74×10^{-5}**	**1.09 (1.04, 1.15)**	**4.42×10^{-4}**	**1.08 (1.02, 1.13)**	**7.20×10^{-3}**

Outcomes	Models	CDI		Domain 1		Domain 2	
		OR (95% CI)	*P*	OR (95% CI)	*P*	OR (95% CI)	*P*
HBP	M1	0.99 (0.96, 1.02)	4.14×10^{-1}	0.99 (0.96, 1.03)	7.03×10^{-1}	0.98 (0.95, 1.01)	1.30×10^{-1}
	M2	1.00 (0.96, 1.03)	8.47×10^{-1}	1.00 (0.97, 1.03)	9.47×10^{-1}	0.99 (0.96, 1.03)	6.68×10^{-1}
Low HDL-C	M1	0.98 (0.95, 1.01)	1.67×10^{-1}	1.00 (0.97, 1.03)	8.69×10^{-1}	**0.94 (0.91, 0.96)**	**4.45×10^{-6}**
	M2	1.00 (0.97, 1.03)	8.56×10^{-1}	1.02 (0.99, 1.05)	1.50×10^{-1}	**0.95 (0.92, 0.98)**	**7.36×10^{-4}**
High TG	M1	**0.93 (0.90, 0.96)**	**4.66×10^{-7}**	**0.95 (0.92, 0.98)**	**8.57×10^{-4}**	0.90 (0.88, 0.93)	9.33×10^{-2}
	M2	**0.96 (0.93, 0.99)**	**2.26×10^{-2}**	0.98 (0.96, 1.02)	3.29×10^{-1}	**0.93 (0.90, 0.96)**	**5.32×10^{-6}**
NHANES Study							
Metabolic syndrome	M1	0.81 (0.78, 0.83)	4.86×10^{-45}	0.78 (0.76, 0.81)	1.23×10^{-57}	0.93 (0.90, 0.95)	2.06×10^{-7}
	M2	0.80 (0.77, 0.84)	2.10×10^{-19}	0.81 (0.78, 0.84)	4.01×10^{-32}	0.93 (0.90, 0.97)	1.49×10^{-4}
Abdominal Obesity	M1	0.83 (0.81, 0.85)	8.75×10^{-45}	0.80 (0.78, 0.82)	6.99×10^{-66}	0.91 (0.88, 0.93)	1.73×10^{-14}
	M2	0.82 (0.79, 0.85)	1.02×10^{-25}	0.80 (0.78, 0.82)	2.06×10^{-57}	0.89 (0.87, 0.92)	4.46×10^{-16}
Hyperglycemia	M1	0.91 (0.87, 0.94)	2.99×10^{-6}	0.91 (0.87, 0.94)	8.81×10^{-7}	0.99 (0.95, 1.03)	7.16×10^{-1}
	M2	0.91 (0.85, 0.98)	1.27×10^{-2}	0.90 (0.86, 0.95)	6.47×10^{-5}	1.01 (0.96, 1.06)	7.70×10^{-1}
HBP	M1	0.92 (0.89, 0.94)	2.07×10^{-8}	0.90 (0.87, 0.92)	7.61×10^{-13}	0.95 (0.92, 0.98)	1.74×10^{-3}
	M2	0.92 (0.86, 0.98)	1.44×10^{-2}	0.92 (0.88, 0.96)	4.96×10^{-4}	0.93 (0.89, 0.98)	4.27×10^{-3}
Low HDL-C	M1	0.87 (0.85, 0.90)	1.69×10^{-21}	0.89 (0.86, 0.91)	7.43×10^{-18}	0.97 (0.95, 1.00)	4.63×10^{-2}
	M2	0.89 (0.86, 0.93)	3.73×10^{-8}	0.91 (0.88, 0.94)	9.06×10^{-11}	1.01 (0.98, 1.04)	3.88×10^{-1}
High TG	M1	0.88 (0.84, 0.92)	2.05×10^{-9}	0.92 (0.88, 0.96)	8.25×10^{-5}	0.94 (0.90, 0.98)	3.65×10^{-3}
	M2	0.92 (0.86, 0.97)	3.50×10^{-3}	0.95 (0.91, 0.99)	2.06×10^{-2}	0.97 (0.93, 1.02)	2.47×10^{-1}

Note: Logistic regression models for z-score standardized CDI and domain scores (independent variable) and metabolism outcomes (dependent variable). M1, model 1, adjusted for age, and sex. M2, model 2, adjusted for age, sex, BMI (not included when abdominal obesity or MetS was outcome), educational attainments, marital status, smoking status, physical activity, antihypertensive medications, lipid-lowering medications, antidiabetic medications, and total energy intake.

[a] Data are presented as ORs (95% CI) and P values.

Abbreviations: CDI, Can-tonese dietary index; CI, confidence interval; GNHS, Guangzhou Nutrition and Health Study; HBP, hypertension; HDL-C, high-density lipoprotein cholesterol; MetS, metabolic syndrome; NHANES, the National Health and Nutrition Examination Survey; OR, odds ratio; TCLSIH, Tianjin Chronic Low-grade Systemic Inflammation and Health; TG, triglycerides.

Table S9. **Association between diseases and two scoring methods of CDI in GNHS and NHANES** [a]

Outcomes	Models	CDI (Quintile)		CDI (Specific quantity)	
		OR (95% CI)	*P*	OR (95% CI)	*P*
GNHS cohort					
Metabolic syndrome	M1	**0.81 (0.75, 0.87)**	**4.32×10^{-9}**	**0.79 (0.74, 0.85)**	**4.21×10^{-10}**
	M2	**0.80 (0.74, 0.86)**	**9.13×10^{-9}**	**0.77 (0.71, 0.83)**	**6.68×10^{-11}**
Abdominal Obesity	M1	**0.92 (0.86, 0.98)**	**8.62×10^{-3}**	**0.87 (0.82, 0.93)**	**7.44×10^{-5}**
	M2	**0.91 (0.85, 0.98)**	**8.35×10^{-3}**	**0.86 (0.79, 0.92)**	**2.98×10^{-5}**
Hyperglycemia	M1	**0.87 (0.78, 0.98)**	**2.10×10^{-2}**	0.93 (0.83, 1.04)	2.16×10^{-1}
	M2	**0.85 (0.74, 0.98)**	**2.31×10^{-2}**	**0.83 (0.72, 0.96)**	**1.32×10^{-2}**
HBP	M1	0.97 (0.90, 1.04)	3.58×10^{-1}	1.01 (0.94, 1.09)	6.97×10^{-1}
	M2	0.96 (0.88, 1.04)	3.17×10^{-1}	0.97 (0.88, 1.06)	4.39×10^{-1}
Low HDL-C	M1	**0.91 (0.85, 0.97)**	**7.25×10^{-3}**	**0.89 (0.83, 0.96)**	**1.95×10^{-3}**
	M2	**0.91 (0.85, 0.98)**	**1.40×10^{-2}**	**0.90 (0.83, 0.97)**	**5.49×10^{-3}**
High TG	M1	**0.90 (0.84, 0.97)**	**4.03×10^{-3}**	**0.90 (0.84, 0.97)**	**4.11×10^{-3}**
	M2	**0.90 (0.84, 0.97)**	**5.21×10^{-3}**	**0.90 (0.83, 0.97)**	**8.19×10^{-3}**
NHANES Study					
Metabolic syndrome	M1	0.81 (0.78, 0.83)	4.86×10^{-45}	**0.87 (0.85, 0.90)**	**3.32×10^{-17}**
	M2	0.80 (0.77, 0.84)	2.10×10^{-19}	**0.85 (0.82, 0.88)**	**3.81×10^{-17}**
Abdominal Obesity	M1	0.83 (0.81, 0.85)	8.75×10^{-45}	**0.83 (0.81, 0.85)**	**9.84×10^{-41}**
	M2	0.82 (0.79, 0.85)	1.02×10^{-25}	**0.81 (0.79, 0.84)**	**1.09×10^{-42}**
Hyperglycemia	M1	0.91 (0.87, 0.94)	2.99×10^{-6}	**1.07 (1.02, 1.11)**	**2.85×10^{-3}**
	M2	0.91 (0.85, 0.98)	1.27×10^{-2}	0.95 (0.89, 1.00)	5.73×10^{-2}

Outcomes	Models	CDI (Quintile)		CDI (Specific quantity)	
		OR (95% CI)	*P*	OR (95% CI)	*P*
HBP	M1	0.92 (0.89, 0.94)	2.07×10^{-8}	0.94 (0.91, 0.97)	5.72×10^{-5}
	M2	0.92 (0.86, 0.98)	1.44×10^{-2}	0.92 (0.88, 0.97)	2.55×10^{-3}
Low HDL-C	M1	0.87 (0.85, 0.90)	1.69×10^{-21}	0.97 (0.94, 1.00)	2.51×10^{-2}
	M2	0.89 (0.86, 0.93)	3.73×10^{-8}	0.97 (0.94, 1.00)	2.84×10^{-2}
High TG	M1	0.88 (0.84, 0.92)	2.05×10^{-9}	0.90 (0.86, 0.94)	4.80×10^{-6}
	M2	0.92 (0.86, 0.97)	3.50×10^{-3}	0.92 (0.88, 0.97)	6.76×10^{-4}

Note: Logistic regression models for z-score standardized CDI derived from two scoring methods (independent variable) and metabolism outcomes (dependent variable). M1, model 1, adjusted for age, and sex. M2, model 2, adjusted for age, sex, BMI (not included when abdominal obesity or MetS was outcome), educational attainments, marital status, smoking status, physical activity, antihypertensive medications, lipid-lowering medications, antidiabetic medications, and total energy intake.

[a] Data are presented as ORs (95% CI) and P values.

Abbreviations: CDI, Can-tonese dietary index; CI, confidence interval; GNHS, Guangzhou Nutrition and Health Study; HBP, hypertension; HDL-C, high-density lipoprotein cholesterol; MetS, metabolic syndrome; NHANES, the National Health and Nutrition Examination Survey; OR, odds ratio; TCLSIH, Tianjin Chronic Low-grade Systemic Inflammation and Health; TG, triglycerides.
